# Allene oxide synthase 1 contributes to limiting grain arsenic accumulation and seedling detoxification in rice

**DOI:** 10.1007/s44154-023-00136-8

**Published:** 2023-11-30

**Authors:** Xin Fan, Haiyang Tang, Xuan Chen, Fanrong Zeng, Guang Chen, Zhong-Hua Chen, Yuan Qin, Fenglin Deng

**Affiliations:** 1 MARA Key Laboratory of Sustainable Crop Production in the Middle Reaches of the Yangtze River, College of Agriculture, Yangtze University, Jingzhou, 434025 China; 2 Central Laboratory, Zhejiang Academy of Agricultural Science, Hangzhou, China; 3 School of Science, Western Sydney University, Penrith, NSW Australia; 4 Hawkesbury Institute for the Environment, Western Sydney University, Penrith, NSW Australia; 5 Hubei Hongshan Laboratory, Wuhan, 430070 China

**Keywords:** Jasmonic acid, Arsenic tolerance, Evolutionary bioinformatics, *Oryza sativa* L., ROS homeostasis

## Abstract

Arsenic (As) is a cancerogenic metalloid ubiquitously distributed in the environment, which can be easily accumulated in food crops like rice. Jasmonic acid (JA) and its derivatives play critical roles in plant growth and stress response. However, the role of endogenous JA in As accumulation and detoxification is still poorly understood. In this study, we found that JA biosynthesis enzymes Allene Oxide Synthases, OsAOS1 and OsAOS2, regulate As accumulation and As tolerance in rice. Evolutionary bioinformatic analysis indicated that AOS1 and AOS2 have evolved from streptophyte algae (e.g. the basal lineage *Klebsormidium flaccidum*) – sister clade of land plants. Compared to other two *AOSs*, *OsAOS1* and *OsAOS2* were highly expressed in all examined rice tissues and their transcripts were highly induced by As in root and shoot. Loss-of-function of *OsAOS1* (*osaos1–1*) showed elevated As concentration in grains, which was likely attributed to the increased As translocation from root to shoot when the plants were subjected to arsenate [As(V)] but not arsenite [As (III)]. However, the mutation of *OsAOS2* (*osaos2–1*) showed no such effect. Moreover, *osaos1–1* and *osaos2–1* increased the sensitivity of rice plants to both As(V) and As(III). Disrupted expression of genes involved in As accumulation and detoxification, such as *OsPT4*, *OsNIP3;2*, and *OsOASTL-A1*, was observed in both *osaos1–1* and *osaos2–1* mutant lines. In addition, a As(V)-induced significant decrease in Reactive Oxygen Species (ROS) production was observed in the root of *osaos1–1* but not in *osaos2–1*. Taken together, our results indicate OsAOS1 modulates both As allocation and detoxification, which could be partially attributed to the altered gene expression profiling and ROS homeostasis in rice while OsAOS2 is important for As tolerance.

**Supplementary Information:**

The online version contains supplementary material available at 10.1007/s44154-023-00136-8.

## Introduction

Arsenic (As) is a natural component of our planet’s crust, which is widely distributed in the environment. The proportion of soil and water contaminated with As was progressively increasing due to anthropogenic activities (Deng et al. 2021; Khanna et al. 2022). For example, out of 112 soil samples in China, 24 (21.4%) had mean soil As concentrations above the World Health Organization (WHO) limit of 30 mg/kg; and a global prediction model shows arsenic exceeding 10 mg/L in most groundwater in Asia, the America and Africa (Antoniadis et al. 2019; Podgorski and Berg 2020). Excessive As is toxic to almost all animals and most plant species. For humans, long-term As intake can cause skin cancer and cancerous changes in internal organs such as the kidney, liver, and bladder (Clemens and Ma 2016). For plants, As toxicity has been observed to reduce photosynthetic pigments in leaves, inhibit plant height, shoot biomass, and reduce tiller number as well as substantial yield losses when five widely cultivated rice accessions were grown in the paddy soil with additional As (Azizur Rahman et al. 2007). The risk resulted from the increasing As concentrations in the soil-plant-human ecosystem is one of the major concerns for global food safety (Guan et al. 2021).

Rice (*Oryza sativa*) is a staple food for more than half of the world population. Arsenite [As(III)] and arsenate [As(V)] are two major forms of inorganic As in soil, which can be absorbed by rice roots through silicic acid transporters and phosphate transporters, respectively (Ma et al. 2008; Cao et al. 2017; Zhao et al. 2022). As is more easily accumulated in rice grains compared to the other cereals such as wheat (*Triticum aestivum*) and barley (*Hordeum vulgare*) partially due to the anoxic growth condition and the highly efficient pathway for silicon absorption (Su et al. 2010; Zhao et al. 2022). A large-scale investigation of a rice core collection consisting of 1763 germplasms revealed that As content in the grains varied from 0.217 to 2.610 mg/kg, with an average of 0.945 ± 0.312 mg/kg (Pinson et al. 2015). In addition, 5 ~ 22% of 471 high-yielding rice varieties also showed high grain As exceeding the threshold even when the plants were grown in the paddy soil with the As content under the national limit (Duan et al. 2017). It was estimated that about 60% of total inorganic As intake in the Chinese population was from the consumption of rice, and the ratio was much higher than the populations in Italy and the United States (Meharg et al. 2009). This indicated that rice is one of the most important sources of inorganic As intake through the food chain for the populations using rice as the staple food (Zhao et al. 2022). Therefore, developing rice lines with reduced grain As is one of the most plausible strategies for reducing human As intake, especially for billions of people in Asia consuming rice as their staple food.

Effective strategies for reducing grain As accumulation through biotechnology had been developed during last decades based on the discovering of the molecular mechanisms on As accumulation in rice (Deng et al. 2019; Deng et al. 2021; Yamaji and Ma 2021; Khanna et al. 2022; Zhao et al. 2022). For instance, overexpression of *Nodulin 26-like Intrinsic Proteins* (*OsNIP1;1* and *OsNIP3;3*) in rice decreased As accumulation in the shoot and grain through disrupting the radial transport of As(III) in roots (Sun et al. 2018). Significantly reduced rice grain As was also succeeded through increasing the expression of *C-type ATP-binding Cassette Transporter 1* (*OsABCC1*) in the root cortical cells together with the enhanced levels of enzymes for phytochelatins (PCs) synthesis (Deng et al. 2018), overexpression of *PC Synthase 1* (*OsPCS1*) (Hayashi et al. 2017), seed-specific silencing of endogenous Multidrug and *Toxic compound Extrusion 2* (*OsMATE2*) (Das et al. 2018), or ectopic expression of *Arsenate Reductase PvACR3;1* from the As-hyperaccumulator, *Pteris vittata* (Chen et al. 2019) and *ScACR3* from yeast (*Saccharomyces cerevisiae*) (Duan et al. 2012). Therefore, enhancing As vacuolar sequestration and chelation, increasing As efflux activity and decreasing As uptake are feasible to reduce As accumulation in rice grains for less human As intake through food chain.

Application of special materials such as fungus, fertilizers, and nanoparticles was also effective for conquering As toxicity and decreasing As accumulation in rice. For example, synergistic application of zinc oxide nanoparticles and salicylic acid in rice through modulation of the cellular redox status and antioxidant defense (Faizan et al. 2021). The colonization of endophytic fungus *Serendipita indica* and phosphorus synergistically recuperate arsenic induced stress in rice (Abbasi et al. 2021; Sehar et al. 2022), mainly through regulating secondary metabolism related enzymatic activity and root metabolic patterns (Sehar et al. 2023a, 2023b).

As a group of critical phytohormone functions in plant growth, development and resistance to environmental stresses (Zhang et al. 2023c), jasmonates [e.g. jasmonic acid (JA), jasmonoyl-l-isoleucine (JA-Ile), and methyl jasmonate (MeJA)] have been implicated in As accumulation and detoxification in plants (Verma et al. 2020; Chen et al. 2021; Li et al. 2022a, 2022b). For example, application of MeJA or JA alleviates As(III) (Mousavi et al. 2020; Verma et al. 2020; Li et al. 2022a, 2022b) and As(V) (Wang et al. 2018; Zhang et al. 2023b) toxicity and accumulation in rice, respectively. Similarly, the effect of MeJA on alleviating As(III) stress in oilseed (*Brassica napus*) was also observed (Farooq et al. 2018). The molecular mechanisms on JA pathway have been extensively studied (Wasternack and Feussner 2018; Hu et al. 2023). Jasmonates are synthesized from α-linolenic acid through the octadecanoid pathway and cytochrome P450 of the CYP74 family enzymes allene oxide synthases (AOSs) are the key enzymes for JA biosynthesis (Wasternack and Feussner 2018). Molecular evolutionary analysis of JA pathway showed that the genes associated with the JA pathway are found in most tested land plants (Chen et al. 2021). There are 4 members of AOS family in the rice genome, which were designated as OsAOS1–4 (Haga and Iino 2004). Purified recombinant OsAOS2 protein expressed in *Escherichia coli* successfully converted 13-hydroperoxylinolenic acid to allene oxid, an important step in the biosynthesis of JA (Ha et al. 2022). Due to the critical roles of JA in resistance to pathogen infection and insect herbivory (Mei et al. 2006; Ye et al. 2013), OsAOSs was widely known as in biotic stresses (Li et al. 2022b). However, the link between the core molecular components in JA biosynthesis and signaling and As tolerance are not well understood in rice.

Therefore, we hypothesized that OsAOSs are key determinates for As accumulation and detoxification in rice plants. We compared the expression pattern of *OsAOSs* among rice tissues and their expression in response to As(III) and As(V), and found that *OsAOS1* and *OsAOS2* showed the highest expression level in the examined tissues, and they were dramatically induced by As. We traced the evolutionary origin of AOS1 and AOS2 in green plants, and the mutant lines of *OsAOS1* and *OsAOS2* were employed for further evaluation of As accumulation and tolerance. Our results suggested that a single amino acid deletion of OsAOS1 increases As sensitivity of the *osaos1–1* plants and higher As accumulation in rice grain.

## Results

### Allene oxide synthases AOS1 and AOS2 are evolutionarily conserved in green plants

**Fig. 1:**
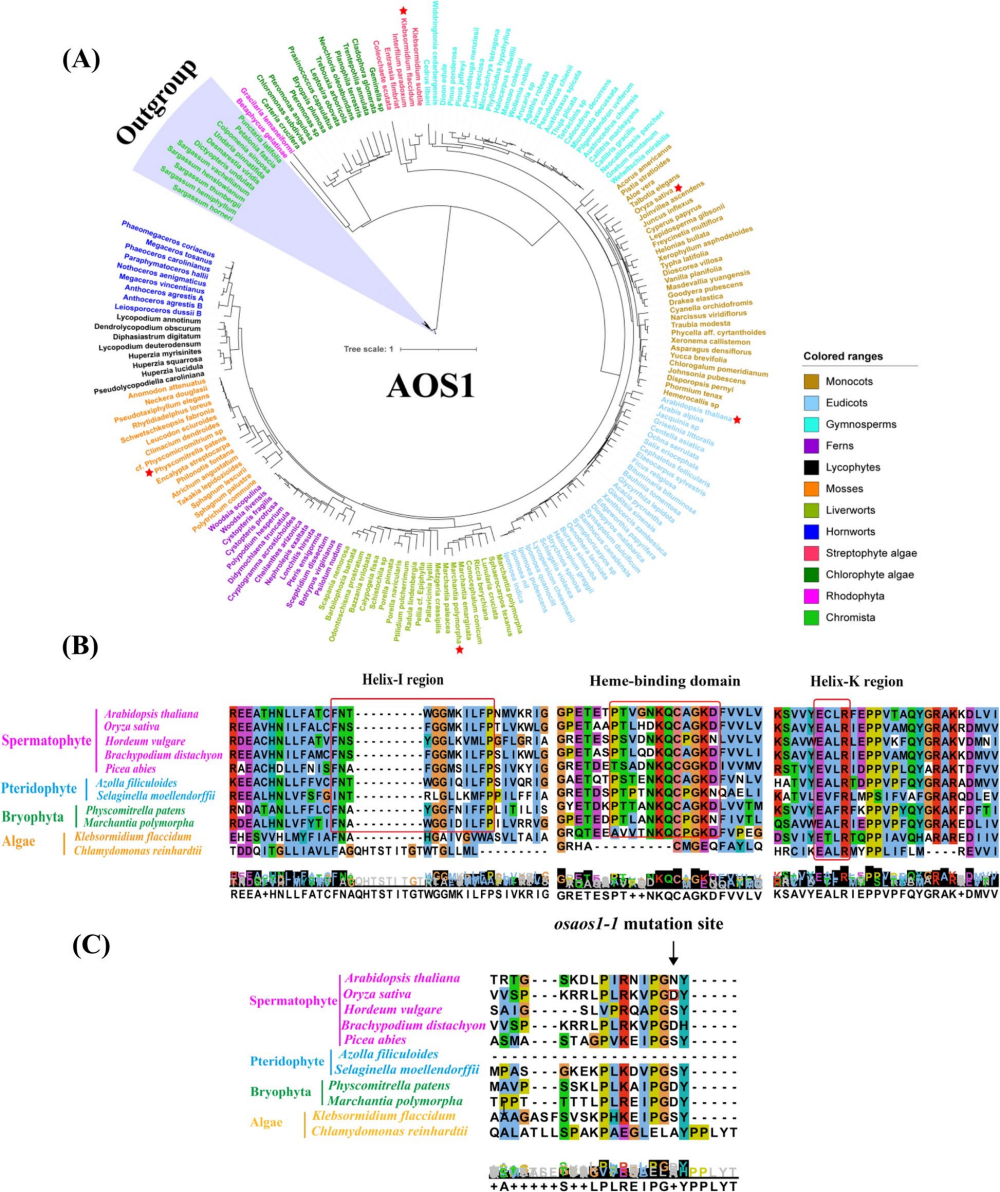
Evolutionary analysis of allene oxide synthase 1 (AOS1) homologues in land plants and algal species. **A** The phylogenetic tree of AOS1 homologues identified from the representative species from various linkages. The orthologues from *Klebsormidium flaccidum*, *Oryza sativa*, *Arabidopsis thaliana*, *Marchantia polymorpha*, *Physcomitrella patens* were highlighted with red asterisks*.* **B** Conserved motifs through multiple protein sequences alignment of AOS1 homologues from selected species. **C** Sequence alignment with the deduced protein fragments containing the mutation site of t of *osaos1–1*

We found that allene oxide synthases required for the biosynthesis of JA are widely presented in land plants, and the homologues of AOSs can be firmly traced to streptophyte algal species (Fig. 1 and S1) such as *Klebsormidium flaccidum*, and possibly from Chlorophyte algae such as *Chlamydomonas reinhardtii* and *Volvox carteri* (Chen et al. 2021). Using the sequence of OsAOS1 as the search query, lots of orthologs were obtained from the OneKP database (One Thousand Plant Transcriptomes Initiative 2019) and 188 orthologs were chosen for the further analysis. The phylogenetic tree of AOS1s from examined plant species showed that AOS1 orthologs could be divided into different clades according to the plant lineages, and the putative orthologues of OsAOS1 from Chromista species were independent of the other subgroups (Fig. 1A). Similar results were also observed when using OsAOS2 as a reference sequence (Fig. S1). It was revealed that PpAOS1 and PpAOS2 from the moss *Physcomitrella patens* (Stumpe et al. 2006), MpAOS1 and MpAOS2 from the liverwort *Marchantia polymorpha*, and KfAOS from *K. flaccidum* (Koeduka et al. 2015) exhibit high amino acid sequence similarity compared to those of angiosperms including rice (Fig. 1 and S1).

Conserved motifs including Helix-I region [GXXX(F/L)], Helix-K region motif (also named as EXLR motif), and the Heme-binding domain (PXVXNKQCPG) of CYP74 members (Chehab et al. 2007; Zhou et al. 2019) were identified in OsAOS1 (Fig. 1B). Further sequence alignment analyses indicated high evolutionary conservation of these domains in the representative species of the major green plant lineages, while the similarity of these domains with those in the chlorophyate alga *C. reinhardtii* was much lower than those from land plants (Fig. 1B). Therefore, AOS1 and AOS2 are largely conserved among green plants in terms of molecular evolutionary aspects.

### *OsAOS1* and *OsAOS2* are highly expressed and induced by As in rice tissues

**Fig. 2:**
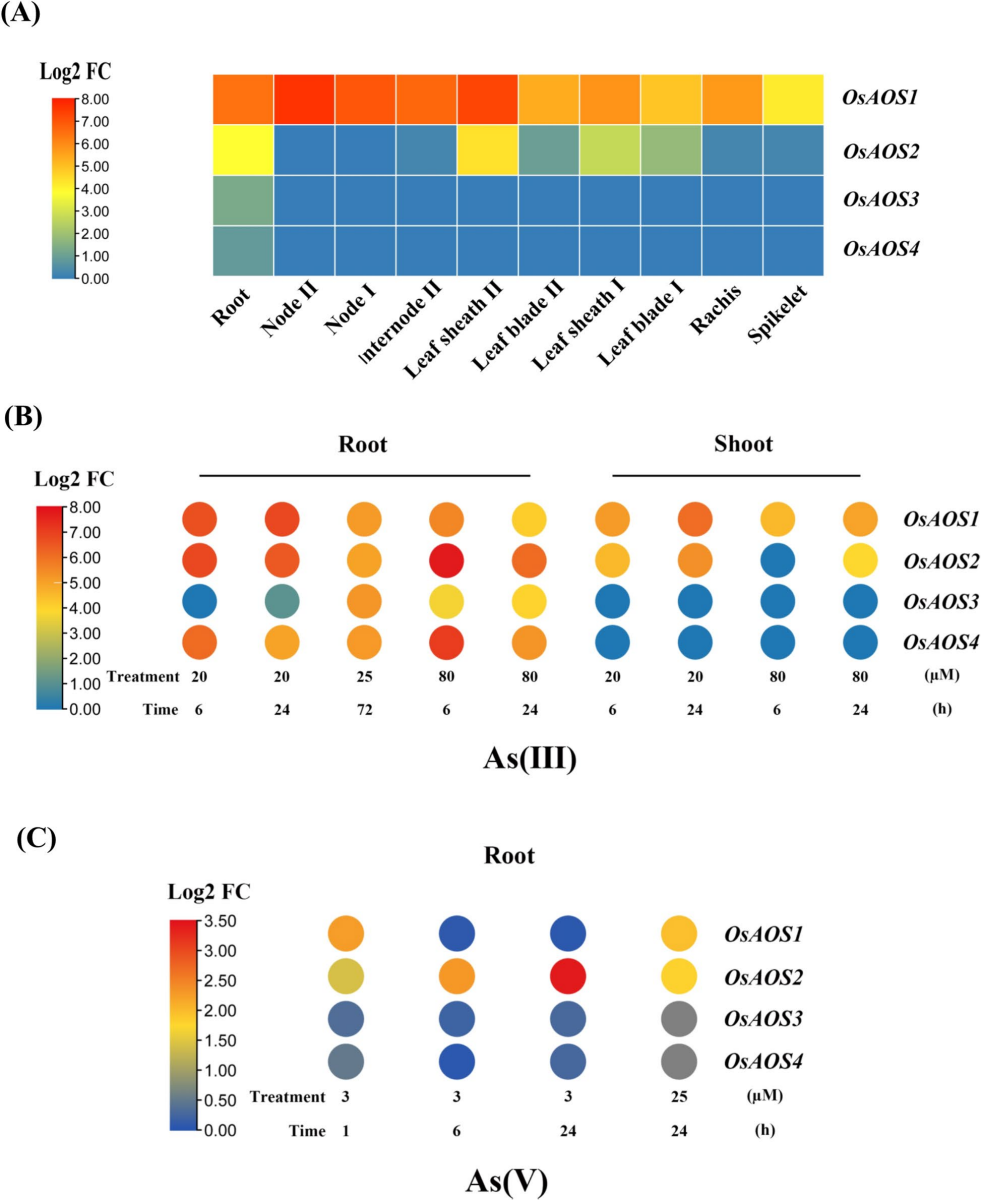
Expression pattern of *allene oxide synthases* (*AOS*) in rice. **A** The relative expression of *OsAOS1–4* in the organs of rice plants cultured in the paddy field harvested at filling stage. The response of *OsAOS1–1* to various condition of As(III) (**B**) and As(V) (**C**)

There are 4 members of AOS family in the rice genome, which were designated as OsAOS1–4 (Haga and Iino 2004). According to the gene expression profiles in various organs of rice plants (*cv.* Nipponbare) grown in paddy soil until filling stage, *OsAOS1* (Os03g0767000, LOC_Os03g55800) displayed constitutively high expression pattern in all examined tissues. The highest expression level of *OsAOS1* was observed in the leaf sheath II, node II, node I, internode II, and root, while moderate transcripts were also detected in the leaf blade and spikelet. Low expression of *OsAOS2* (Os03g0225900, LOC_Os03g12500) was observed in the nodes, internode, rachis and spikelet. *OsAOS3* (Os02g0218700, LOC_Os02g12680) and *OsAOS4* (Os02g0218800, LOC_Os02g12690) showed low expression levels except in roots (Fig. 2A).

The response of *OsAOSs* to As(V) and As(III) was further compared by using published transcriptomic datasets (Huang et al. 2012; Yu et al. 2012; Huang et al. 2019). Dramatically induced expression of *OsAOS1–4* was observed in the roots of plants in response to 20, 25 or 80 μM As(III) for 6 h to 72 h, while transcripts of *OsAOS3* and *OsAOS4* in the shoots were not responsive to As(III) treatment (Fig. 2B). The enhanced expression of *OsAOS1* in the root tip (0–2 cm of *cv.* Zhonghua 11) was observed with 3 μM As(V) treatment for 1 h but rapidly decreased at 6 h and 24 h of As(V) treatment. On the other hand, the transcripts of *OsAOS2* were increased progressively along with the treatment time under 3 μM As(V) (Fig. 2C). Under the treatment of 25 μM As(V), significantly increased transcripts of *OsAOS1* and *OsAOS2* in roots were also detected, but the expression of *OsAOS3* and *OsAOS4* was hardly affected by As(V) (Fig. 2C). All these results indicated that two key enzymes for JA biosynthesis OsAOS1 and OsAOS2 respond to both As(III) and As(V) in rice, rendering further functional analysis.

### Mutation of *OsAOS1* increases grain As accumulation

To investigate the molecular function and physiological role of *OsAOS1* and *OsAOS2*, the gene-edited lines generated through clustered regularly interspaced short palindromic repeats/CRISPR–associated nuclease 9 (CRISPR/Cas9) were obtained from the commercial rice genome-scale mutagenesis library (Lu et al. 2017). The target sites were in the exons of *OsAOS1* and *OsAOS2*, respectively (Fig. S2A, S3A). Sequencing results identified a 3-bp deletion (c.156_158delGGA) and a 1-bp insertion plus a substitution from G by T (c.157G > T + c.158inT) in the offspring of T2 generation of *OsAOS1* mutant lines, leading to an amino acid (p.Asp53del) deletion or frameshift-induced pre-stop (p.Asp53TyrfsTer152), respectively (Fig. S2B). The mutants were then designated as *osaos1–1* and *osaos1–2*. Truncated OsAOS2 was observed in the mutant line *osaos2–1* (p. Pro58AlafsTer424)*,* which was resulted from a 1-bp insertion (c.171inC) in the coding region and corresponding frameshift (Fig. S3B). The deleted amino acid Asp(D)53 in *osaos1–1* was conserved with the putative homologues of AOSs from *Brachypodium distachyon*, *Physcomitrella patens*, and *Marchantia polymorpha* but not in *Arabidopsis thaliana* or *Hordeum vulgare* (Fig. 1C), and the predicted 3D structure of this mutant was almost identical to that of the functional OsAOS1 (Fig. S2C). In addition, the mutation site of *osaos2–1* occurred in a putative conserved domain among land plants (Fig. S1B), and the predicted 3D structure also confirmed the large fragment deletion (Fig. S3C).

**Fig. 3:**
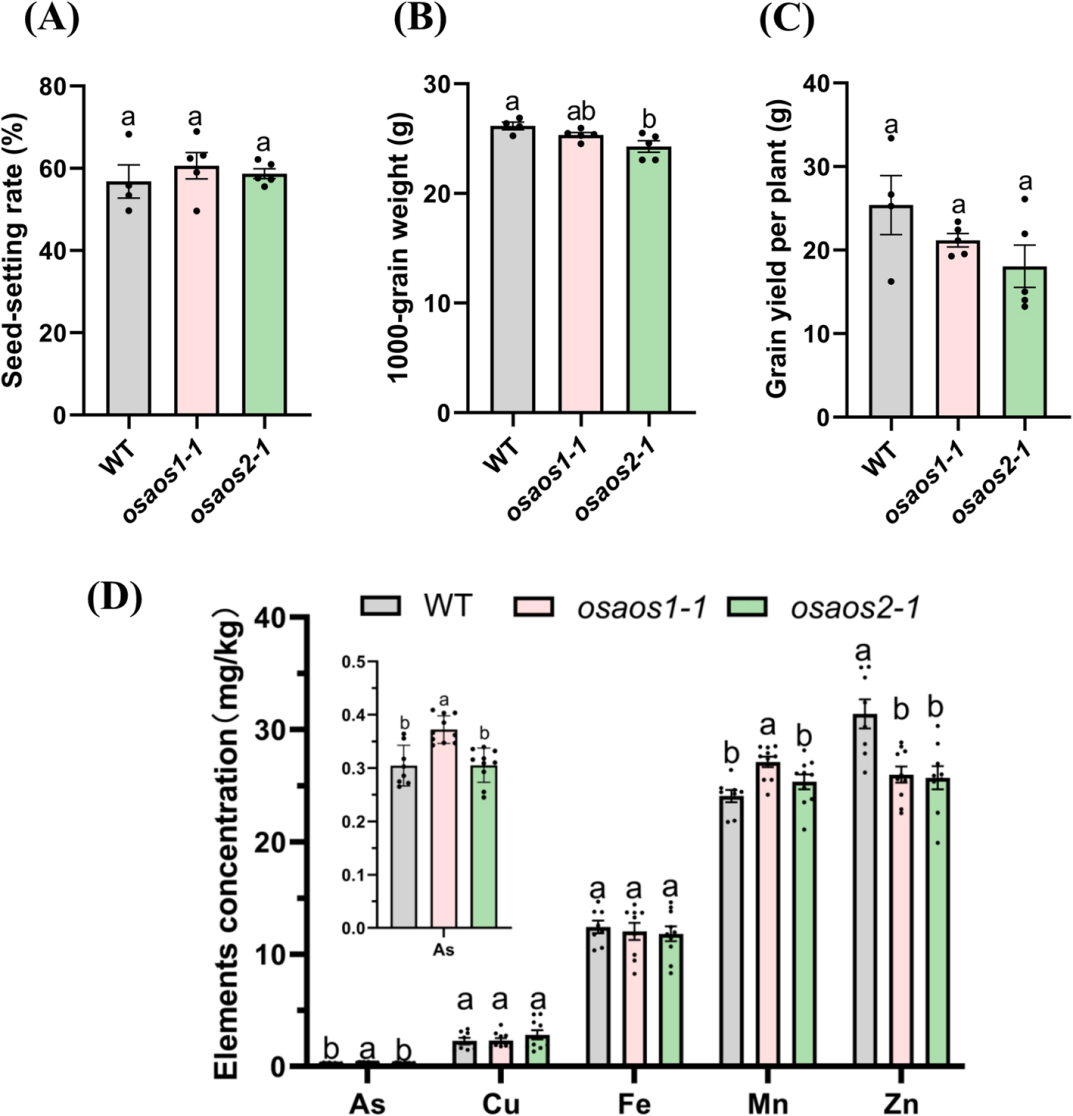
Yield-related traits and mineral concentrations in the grain of *osaos1–1*, *osaos2–1* and WT. Seed setting rate (**A**), grain weight (**B**) and grain yield per plant (**C**) of *osaos1–1*, *osaos2–1* and WT grown in soil-contained pots until mature. **D** The concentrations of As, Cu, Fe, Mn and Zn in the brown rice of the mutant lines and WT. Values are means of three independent replicates ± SE (*n* = 4 for A-C, and *n* = 10 for D). Different letters indicate significant differences in each graph (*p* < 0.05)

Due to the extremely low seed setting of *osaos1–2,* the homozygous mutant lines, *osaos1–1* and *osaos2–1* were selected for the further analysis. Five independent plants of each mutant line and the corresponding wild-type plants (WT, *cv.* Zhonghua 11) were grown to maturity in pots with paddy soil. There was little variation on the seed setting rate, grain weight between the mutant lines and the WT plants (Fig. 3A, B). Grain yield per plant of *osaos1–1* and *osaos2–1* was slightly lower (not significant *P* > 0.05) than that of WT (Fig. 3C). In comparison with WT, significant increase of As and manganese (Mn) concentrations were found in the brown rice grains of *osaos1–1,* while there was no significant difference in the concentrations of iron (Fe), and copper (Cu) between WT and *osaos1–1* (Fig. 3D). Significant decrease of Zn concentration was observed in the brown rice of *osaos1–1* and *osaos2–1*, while mutation of *OsAOS2* hardly affected the accumulation of the other examined metals (Fig. 3D). The results indicated that the deletion of Asp53 in OsAOS1 hardly affect the yield but significantly increase As accumulation in rice grain.

### Mutation of *OsAOS1* increases As translocation from root to shoot

**Fig. 4:**
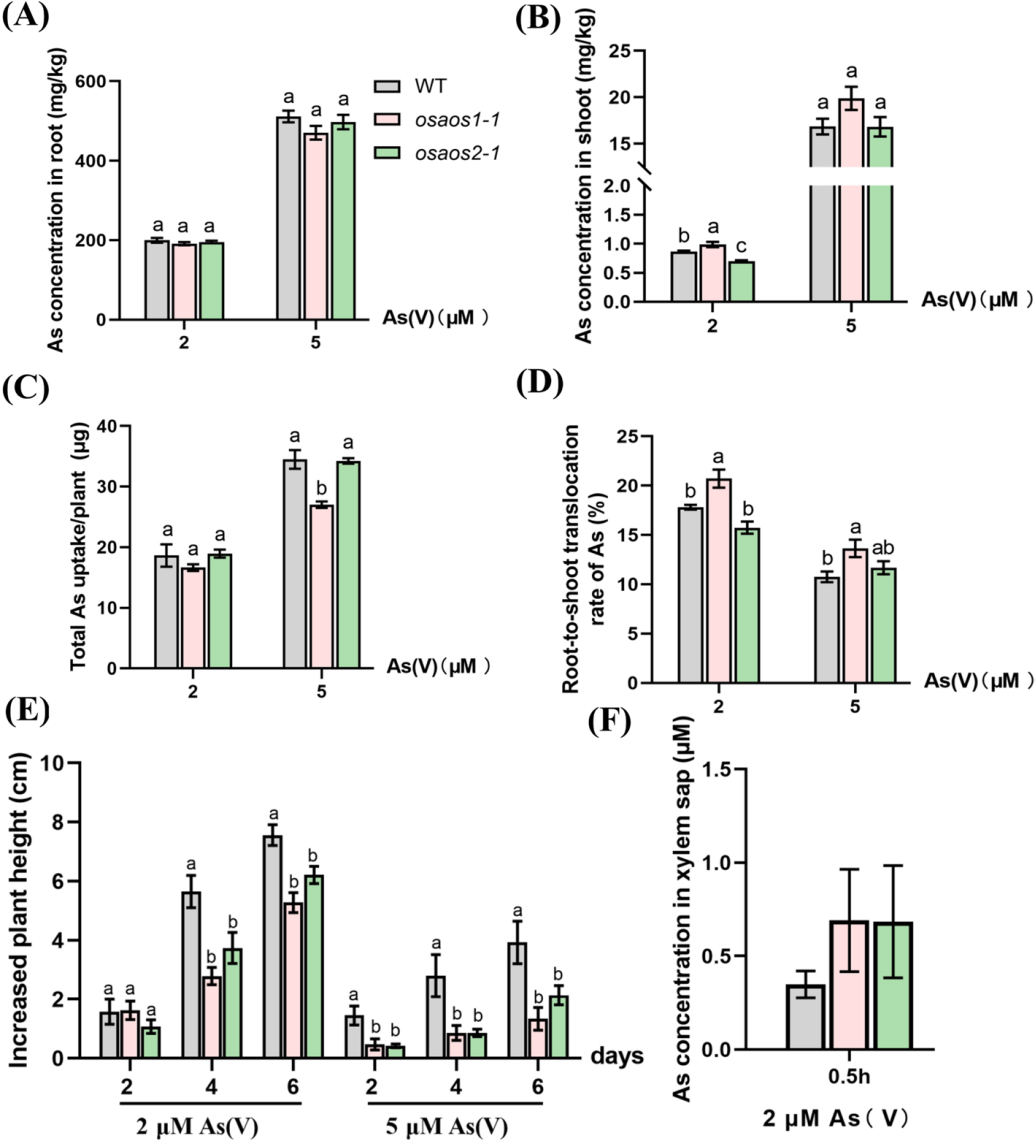
As accumulation and distribution in the seedlings of *osaos1–1*, *osaos2–1* and WT at vegetative growth stage. As concentration in the root (**A**), shoot (**B**) of the plants harboring 3–4 leaves subjected to 2 or 5 μM As(V) for 6 days. Total As taken up by the plants (**C**) and the ratio of As translocated from root to shoot of each genotype (**D**) was calculated. **E** Increased plant height before and after the As treatments. **F** As in the xylem sap collected from the 30-day-old plants treated with 2 μM As(V) for 0.5 hour. Values are means of three independent replicates ± SE (n = 4). Different letters indicate significant differences in each graph (*p* < 0.05)

When the plants were treated with 2 and 5 μM As(V) for 6 d, no difference of As concentrations in the root of the plants was observed (Fig. 4A), however, significantly increased As concentration was found in the shoots of *osaos1–1* compared to that of WT in 2 μM As(V) (Fig. 4B). Significantly lower total As was taken up by the roots and accumulated in the whole plants of *osaos1–1* than that of WT (Fig. 4C), while the ratio of As translocated from root to shoot in *osaos1–1* was significantly higher than that of WT (Fig. 4D). Xylem sap As concentration was higher (no statistical significance) in *osaos1–1* and *osaos2–1* than that of WT subjected to 2 μM As(V) for 30 min (Fig. 4F). However, mutation of *OsAOS2* hardly affected As distribution between root and shoot (Fig. 4).

As concentrations were significantly decreased in the roots of *osaos1–1* and *osaos2–1* compared to that of WT when the plants were cultured with 2 or 5 μM As(III) for 6 d (Fig. S4A). The total As uptake by the mutant lines was also much lower than that of WT (Fig. S4C). However, there was no difference in shoot As concentration and As distribution ratio between root and shoot of the three genotypes (Fig. S4B, D). We then compared As efflux activity from root to external solution after the 15-d-old plants in short-term (4 h) As(V) treatment. After transferring to the nutrient solution without As for 6 h, As efflux percentage (%) was calculated according to the As accumulated in plant root, shoot and presented in the external solution. The results revealed that mutation of *OsAOS1* or *OsAOS2* did not affect As efflux activity (Fig. S5). Thus, our results suggested that increased grain As in *osaos1–1* mutant is likely attributed to the elevated As translocation from root to shoot subjected to As(V).

### OsAOS1 and OsAOS2 are the key components of As tolerance in rice

When the plants were subjected to As(V) treatment, the growth of WT and both *aos* mutant lines were significantly affected (Fig. S6). Compared to the WT, remarkably lower increased plant height in both *osaos1–1* and *osaos2–1* was observed after the treatment for 4 d in 2 μM As(V), while the difference could be observed in the treatment with 5 μM As(V) from 2 d (Fig. 4E). The root and shoot dry weights *osaos1–1* were significantly lower than those of WT, but there was no difference between *osaos2–1* and WT in terms of biomass (Fig. S6A, B). Interestingly, the root and shoot dry weights displayed no difference among *osaos1–1, osaos2–1* and WT subjected to 2 or 5 μM As(III) for 6 d (Fig. S7). It’s implicated that *osaos1–1* and *osaos2–1* are more sensitive to As(V) than As(III).

**Fig. 5:**
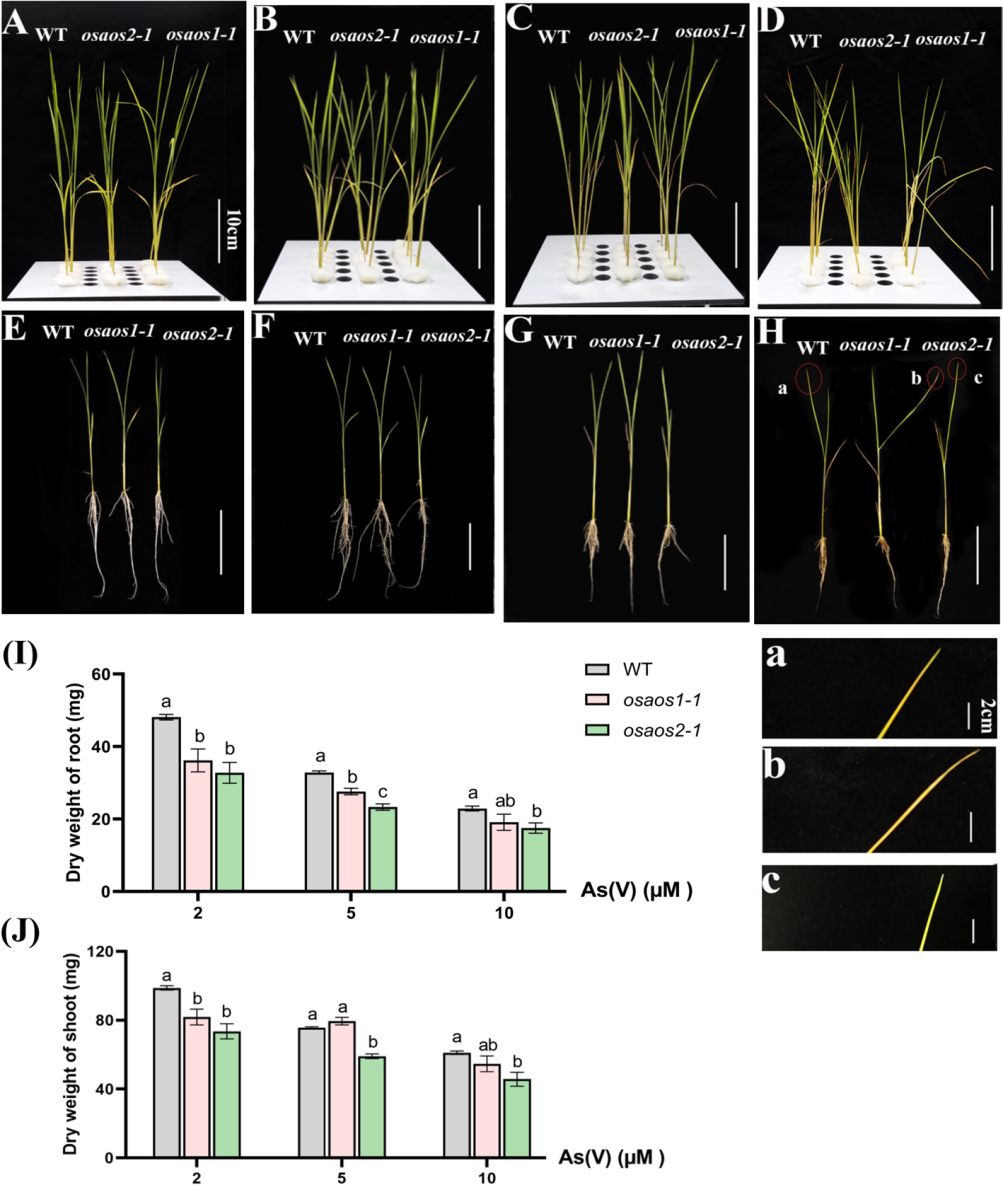
Plant growth and biomass of *osaos1–1*, *osaos2–1* and WT under As(V) treatment. Ten-day-old seedlings of the three genotypes subjected to 0 (**A**, **E**), 2 (**B**, **F**), 5 (**C**, **G**) or 10 μM As(V) (**D**, **H**) for 10 days. The aboveground tissues (**A**-**D**) and whole plants (**E**-**H**) were recorded, and the youngest leaves of the plants under 10 μM As(V) were zoomed in a-c. The dry weight of root (**I**) and shoot (**J**) of the plants were measured. Values are means of three independent replicates ± SE (n = 4). Different letters indicate significant differences in each graph (*p* < 0.05)

Compared to those of WT, curly and chlorosis young leaves, and incompact plant architecture were more clearly presented in *osaos1–1* under the highest As(V) condition (Fig. 5A-H, a-c). In addition, significantly reduced root and shoot dry weights were observed in the mutant lines. Particularly at 2 μM As(V), root dry weight was decreased by 25% and 32% in *osaos1–1* and *osaos2–1* while shoot dry weight was reduced by 17% and 26% in *osaos1–1* and *oasos2–1*, respectively (Fig. 5I, J).

**Fig. 6:**
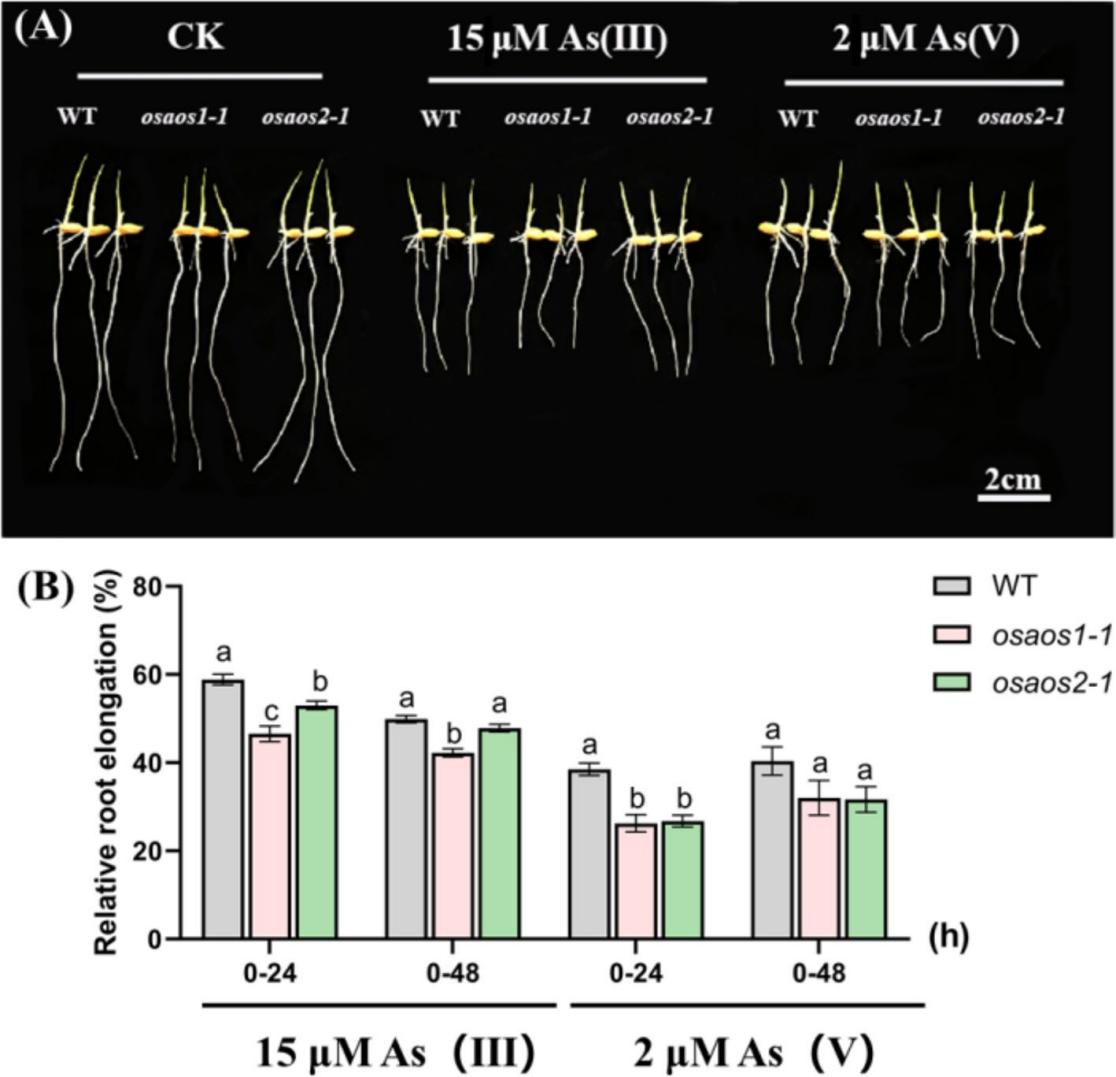
Relative root elongation of *osaos1–1*, *osaos2–1* and WT subjected to As. Two-day-old seedlings of the plants were treated with 15 μM As(III) or 2 μM As(V) for 2 days, the length of the seminal root was measured (**A**) and the relative root elongation rate was calculated (**B**). Values are means of three independent replicates ± SE (*n* = 10). Different letters indicate significant differences in each graph (*p* < 0.05)

The root length and relative elongation of seminal roots of the three genotypes under As treatments showed no difference under control condition, but both were significantly reduced root length in *osaos1–1* subjected to 15 μM As(III) or 2 μM As(V) for 2 d (Fig. 6A and B). The retarded root growth was also found when *osaos2–1* was cultured in the presence of As(III) and As(V) for 24 h (Fig. 6B), but the inhibition of root elongation by As(III) was only statistically significant at 0–24 h (Fig. 6B).

### Disrupted gene expression in the mutant lines of *OsAOS1* and *OsAOS2*

**Fig. 7:**
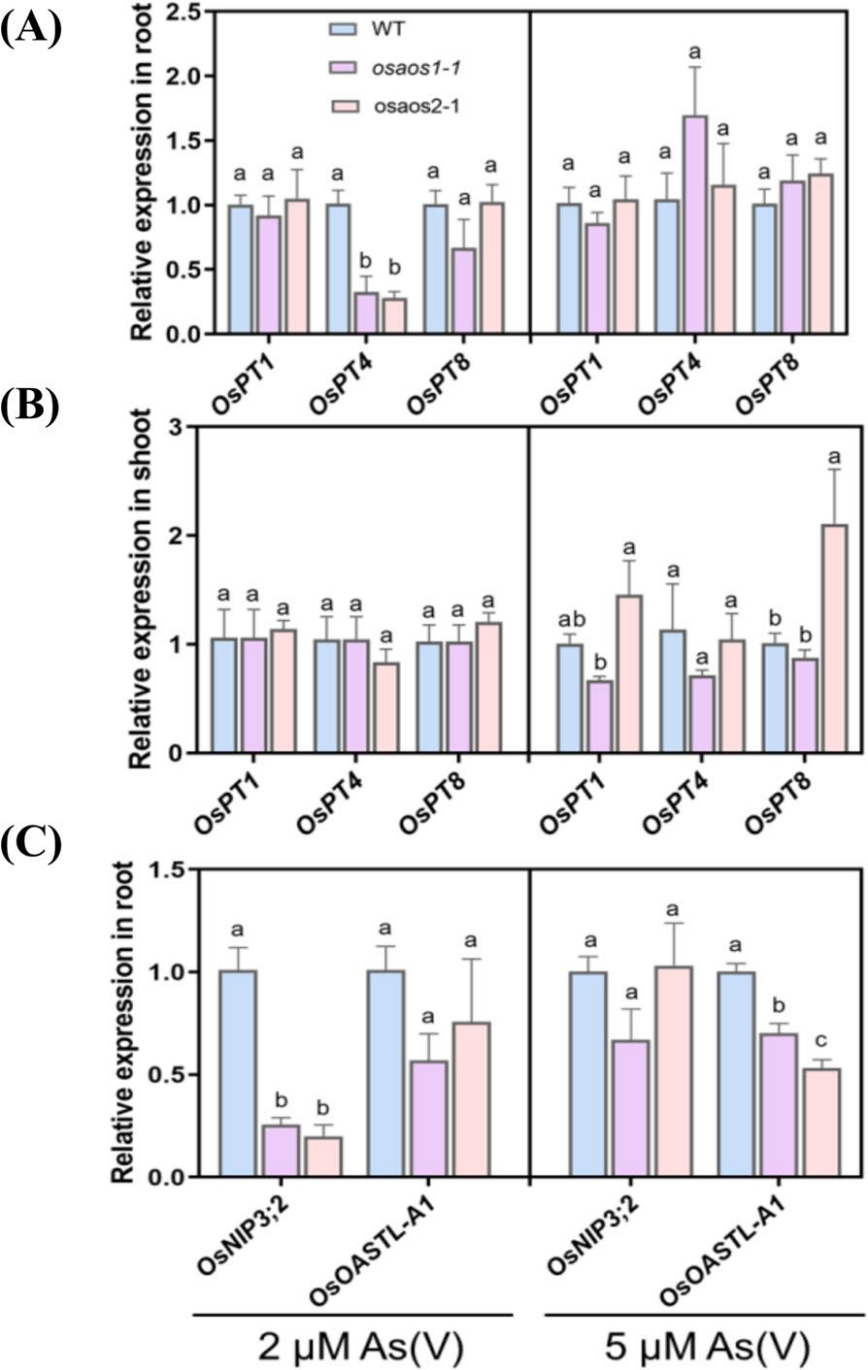
Expression of genes involved in As accumulation and detoxification in rice. After being treated with 2 or 5 μM As(V) for 6 days (Same condition as that in Fig. 4A-D), the root and shoot were harvested for RNA extraction and 1-st strand cDNA synthesis. The relative expression levels of the genes were compared through quantitative real-time PCR. Values are means of three independent replicates ± SE (*n* = 3). Different letters indicate significant differences in each graph (*p* < 0.05). Abbreviations: *Phosphate (P) transporters* (*OsPT1,OsPT4, OsPT8*)*, Nodulin 26-like Intrinsic Protein* (*OsNIP3;2*)*, O-acetylserine* (*thiol*) *lyase* (*OsOASTL-A1*)

To dissect the potential molecular mechanisms underlying the above observations, the expression levels of the critical genes involved As accumulation and detoxification mediated by OsAOS1 and OsAOS2 were determined. Among the genes for As(V) uptake including several *Phosphate (P) transporters* (*OsPT1 OsPT4*, and *OsPT8*), upregulated expression of *OsPT8* was observed in the shoots of *osaos2–1* and dramatic downregulation of *OsPT4* was found in the root of both *osaos1–1* and *osaos2–1* in the presence of 2 μM As(V) for 6 d (Fig. 7A, B). Significant down-regulated expression of *OsNIP3;2* was also observed in the root of the two mutant lines (Fig. 7C). Significant upregulation of *Low Silicon 6* (*OsLsi6*) was induced by 2 μM As(V) in the shoot and 5 μM As(V) in the root of *osaos1–1*. However, expression of *OsLsi1*, *OsLsi2*, *OsLsi3*, *OsABCC1*, *OsABCC7*, *OsHAC1;1*, *OsHAC1;2,* and *OsHAC4* remained unchanged among the genotypes in response to As(V) treatments (Fig. S8). Significant downregulation of *OsOASTL1-A1* involved in As detoxification was identified in the root of both mutant lines subjected to 2 μM As(V) compared to that of WT (Fig. 7C). As a result, the altered expression of the genes involved in As accumulation and tolerance was obvious in the mutant lines of *OsAOS1* and *OsAOS2*.

### Decreased reactive oxygen species (ROS) accumulation in the root of *osaos1–1*

**Fig. 8:**
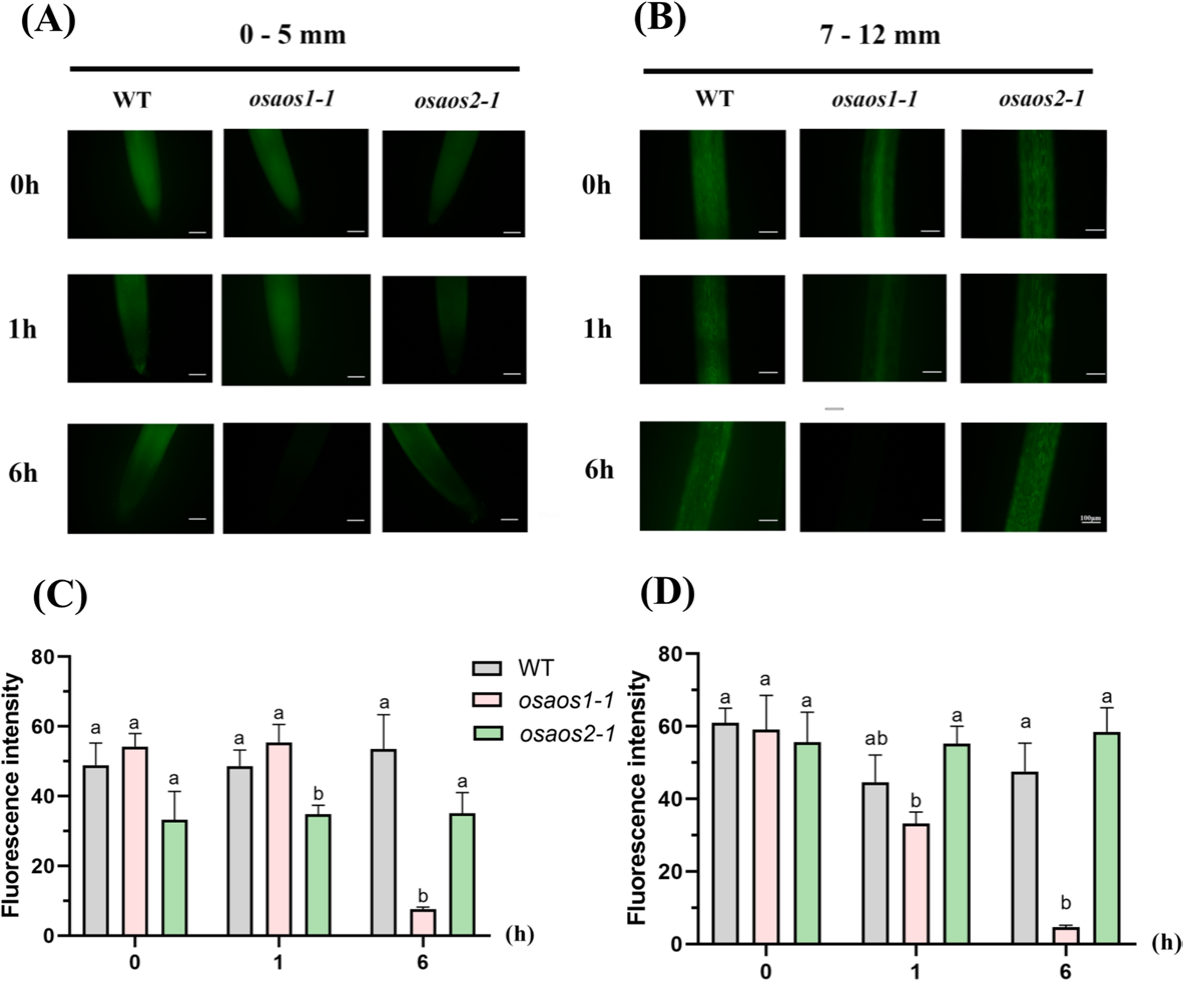
ROS accumulation in the roots of *osaos1–1*, *osaos2–1* and WT subjected to As(V). After being treated with 2 μM As(V) for 1 or 6 hours, the seminal root of the 3-day-old seedlings were stained with 10 μM CM-H_2_DCFDA for 1 hour and was washed for 3 times was employed for ROS detection. The representative figures were present in (**A**) and (**B**). The signal intensity of root tip (0–5 mm) (**C**) and basal root region (7–12 mm) (**D**) was calculated from 5 independent replicates. Values are means of three independent replicates ± SE (*n* = 5). Different letters indicate significant differences in each graph (*p* < 0.05). Scale bar = 100 μm

ROS can be rapidly induced by 25–400 μM As in plants (Deng et al. 2020; Sharma et al. 2021), and MeJA alleviates As toxicity in rice partially through ROS scavenging (Lu et al. 2020). These shed some light on the potential involvement of OsAOS1 and OsAOS2 in As detoxification through ROS homeostasis. It was found that no significant difference in ROS fluorescence was observed in the root tip (0–5 mm) and basal root region (7–12 mm) of WT exposed to 2 μM As(V) for 1 and 6 h. There was no difference in ROS production with 1 hour As(V) treatment between *osaos1–1* and WT (Fig. 8) However, the fluorescent signal intensity was dramatically decreased in both root tip and basal root region of *osaos1–1* in response to 2 μM As(V) for 6 hours (Fig. 8). Interestingly, mutation of *OsAOS2* hardly affected the accumulation of ROS in root in both control and As(V) treatment (Fig. 8). The result indicated that mutation of *OsAOS1* but not *OsAOS2* showed disrupted ROS homeostasis, which could be related to a higher root to shoot As translocation rate in *osaos1–1* mutant.

## Discussion

### OsAOS1 is involved in As accumulation in rice

Arsenic contamination in plant derived-food is a worldwide health concern (Zhao et al. 2022). Several pathways for phytohormone metabolism and signaling transduction have been identified in response to As stress (Hu et al. 2020; Chen et al. 2021; Deng et al. 2021; Sharma et al. 2021; Li et al. 2023; Zhang et al. 2023a). It was reported that the transcripts of key genes involved in JA biosynthesis and signaling are significantly regulated by As(III) and As(V) in rice, barley, and Arabidopsis (Huang et al. 2012; Yu et al. 2012; Srivastava et al. 2015; Zvobgo et al. 2018). Application of exogenous JA partially alleviated As(V)-induced rice root inhibition, and pretreated with JA decreased As accumulation in both shoot and root of rice seedlings subjected to As(V) (Wang et al. 2018; Ronzan et al. 2019; Mousavi et al. 2020; Li et al. 2022a, 2022b; Zhang et al. 2023b). These results implicated the involvement of JA in As accumulation and detoxification in plants. However, whether As translocation and tolerance were controlled by JA biosynthesis and signaling is poorly understood. Here, we demonstrated that OsAOS1, a putative enzyme for JA biosynthesis, limits grain As accumulation (Fig. 3D). In addition, the contribution of OsAOS1 to As accumulation in rice was preferentially more effective when subjecting plants to As(V) instead of As(III) (Fig. 4, S4).

The physiological processes and some critical proteins involved in As accumulation in rice grain and As distribution among various tissues have been revealed recently (Deng et al. 2020; Deng et al. 2021; Tang and Zhao 2021; Yamaji and Ma 2021; Zhao et al. 2022). Briefly, the uptake and translocation from root to shoot of arsenous acid [As(OH)_3_] is achieved by the effective cooperation of two silicon (Si) transporters, OsNIP2;1 (OsLsi1, low silicon rice 1), and OsLsi2 (Ma et al. 2008). The homologues of OsLsi1, OsNIP3;2, is involved in As(III) uptake by lateral roots (Chen et al. 2017). The aquaporins including OsLsi1, OsNIP1;1 and OsNIP1;3 were also responsible for the efflux of As(III) (Zhao et al. 2010; Sun et al. 2018), which contribute to As accumulation. The tonoplast-localized C-type ATP-binding cassette transporter OsABCC1 plays critical roles in limiting As accumulation in rice grain and in As detoxification through sequestration of As-phytochelatins (As-PCs) into vacuole (Song et al. 2014; Deng et al. 2018), while OsABCC7 is involved in the root-to-shoot translocation of As in rice (Tang et al. 2019). Phosphate (P) transporters OsPT1, OsPT4 and OsPT8 also function in the uptake of As(V) and accumulation of As, and the contribution of OsPT4 in the inorganic As accumulation in rice grain was estimated as 20 ~ 44% (Kamiya et al. 2013; Wang et al. 2016; Cao et al. 2017), which implicated that As(V) could be considered as a major source of As contamination for rice grown in anoxic condition. Furthermore, the As(V) absorbed by rice roots can be readily transformed to As(III) in the presence of arsenate reductases including OsHAC1;1, OsHAC1;2 and OsHAC4, which also contribute to the accumulation and detoxification of As in rice plants (Shi et al. 2016; Xu et al. 2017).

Here, we found mutation of *OsAOS1* significantly increased As content in the brown rice grains of the plants grown in soils with additional As (Fig. 3D), which was probably through increasing As translocation from root to shoot but not via As uptake and efflux (Fig. 4 and Fig. S5). Interestingly, the increased As translocation from root to shoot was only observed in *osaos1–1* exposed to As(V) but not to As(III) (Fig. 4 and Fig. S4). The results suggested that the contribution of OsAOS1 to As accumulation was likely dependent on the pathway of phosphate (P) but not silicic acid accumulation, which was consistent with the altered expression of *OsPT1*, and *OsPT4* in *osaos1–1* (Fig. 7). While the expression of genes including *OsLsi1*, *OsLsi2*, *OsLsi3* and *OsLsi6* responsible for Si uptake, root-to-shoot translocation and distribution was hardly affected except the increased transcription of *OsLsi6* in *osaos1–1* (Fig. S8). The expression of *OsHAC1;1*, *OsHAC1;2*, and *OsHAC4* was hardly affected by the mutation of OsAOS1 or OsAOS2 (Fig. S8), implicating that the deoxidize of As(V) to As(III) in rice root cells was likely not affected by JA pathway.

### The role of OsAOS1 in alleviating As toxicity in rice

To adapt to the environmental stresses, plants have evolved various strategies to prevent themselves from the damage. JA and its derivatives represent a group of phytohormones, eliciting various effects on the tolerance of plant to both biotic and abiotic stresses (Yu et al. 2019). Pretreatment with JA or MeJA alleviates As toxicity in rice, especially in root growth (Wang et al. 2018; Ronzan et al. 2019; Mousavi et al. 2020). Consistently, enhanced sensitivity including inhibited root elongation and withered young leaves was observed in *osaos1–1* and *osaos2–1* subjected to both As(III) and As(V) compared to that of WT (Figs. 5, 6 and Fig. S6), indicating that OsAOS1 and OsAOS2 may confer to As detoxification.

The alleviated effects mediated by JA was widely observed in plants subjected to cadmium (Cd), salt, drought and other abiotic stresses (Yu et al. 2019; Kamiya et al. 2013; Wang et al. 2020). Consistent with the rapidly induced expression of *OsAOS1* and *OsAOS2* in rice (Fig. 1B, C) (Huang et al. 2012; Yu et al. 2012), the expression of genes promoting endogenous JA synthesis was also upregulated by Cd treatment in Arabidopsis (Lei et al. 2020). Exogenous application of MeJA partially alleviated Cd-generated chlorosis of new leaves (Lei et al. 2020). Consistently, mutation of *OsAOS1* or *OsAOS2* enhanced the curling and chlorosis of young leaves (Fig. 5). Increased shoot As and reduced root As concentrations were observed in the rice mutant *coleoptile photomorphogenesis2* (*cpm2*), the loss-of-function of *Allene Oxide Cyclase* (*AOC*) required for JA synthesis (Ronzan et al. 2019), which was very similar to our finding in *osaos1–1* (Fig. 4). Furthermore, the phenotype of retarded root system of *cpm2* exposed to As is similar to that of *osaos1–1 and osaos2–1* (Figs. 5 and 6) (Ronzan et al. 2019). Knockout of *AtAOS* increased Cd concentration in both roots and shoots, and confered to the increased sensitivity to Cd, which could be recovered by exogenous MeJA (Lei et al. 2020). The expression of genes responsible for Cd uptake and long-distance transport such as *AtIRT1, AtHMA2* and *AtHMA4* was greatly upregulated in *ataos* mutant but decreased in the presence of MeJA (Lei et al. 2020), however, only the translocation of As from root to shoot but not the uptake and efflux of As was not affected in *osaos1–1* (Fig. 4, S5)*.*

ROS homeostasis in plant cells also confers the detoxification of heavy metals (Huang et al. 2012; Smirnoff and Arnaud 2019; Nazir et al. 2020). Application of exogenous ROS also decreased the accumulation of heavy metals in root through reducing root uptake and facilitating root-to-shoot translocation of heavy metals (Cheng et al. 2023; Deng et al. 2023). It’s implicated that the ameliorating effects of JA partially rely on ROS scavenging (Chen et al. 2021). In this study, dramatically decreased ROS was detected in the mutant lines of *OsAOS1* but not *OsAOS2* in response to As(V) (Fig. 8), which might be one of the possible strategies for As detoxification mediated by OsAOS1. The reason for the increased As sensitivity of *osaos2–1* is probably attributed to the reduced expression level of *OsABCC1* and *OsOASTL-A1* (Fig. 7, S8).

Synergistic interplay of one of the major ROS - hydrogen peroxide (H_2_O_2_) with JA was found in plants. For example, application of MeJA induced accumulation of H_2_O_2_ in the detached leaves of rice (Hung et al. 2006), while H_2_O_2_ could act as a signaling molecule for the MeJA-triggered antioxidant defense in wheat (Nazir et al. 2020). H_2_O_2_ at nanomolar levels is helpful in the maintenance of cellular homeostasis in crops, while elevated levels of H_2_O_2_ trigger oxidative burst and lead to cell death (Smirnoff and Arnaud 2019). Therefore, the production, intracellular transport and scavenging of ROS should be tightly regulated. In this study, ROS accumulation was inhibited in the root of *osaos1–1* subjected to As(V) compared to that of WT (Fig. 8), which is probably attributed to the increased As translocation from root to shoot (Fig. 4).

**Fig. 9:**
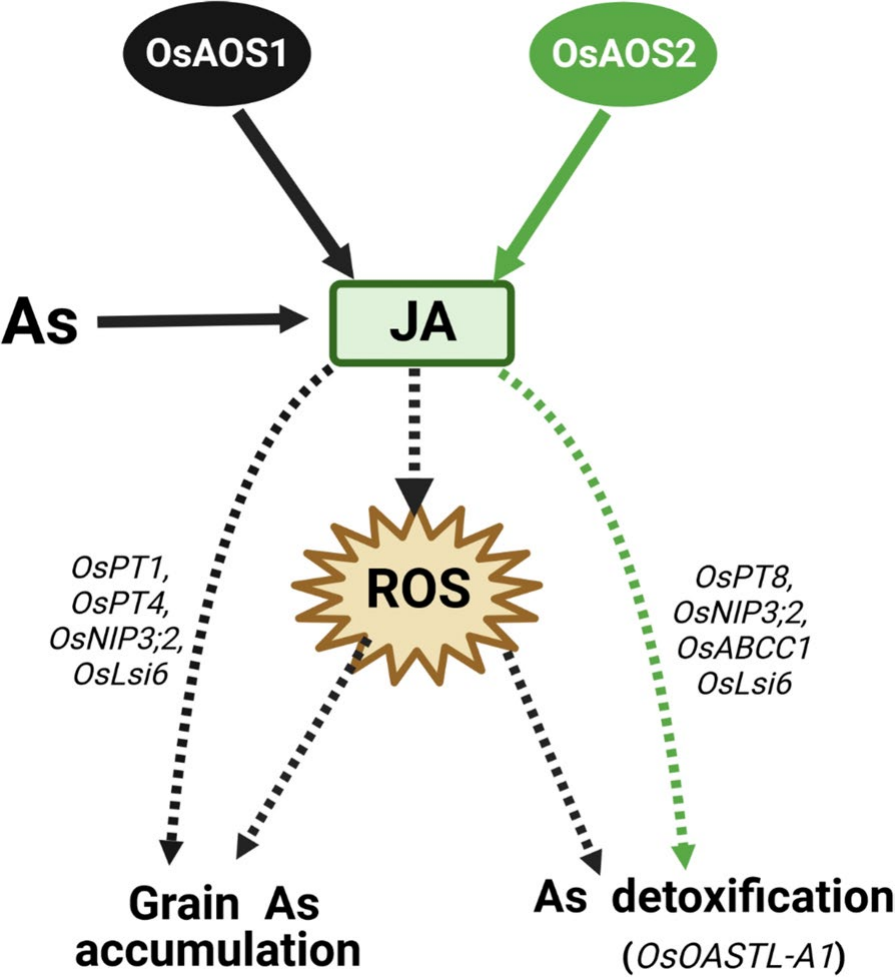
OsAOS1 and OsAOS2 contribute to differential accumulation and detoxification of As in rice. OsAOS1 (black routes) and OsAOS2 (green routes) are the key enzymes for the biosynthesis of JA in rice. OsAOS1 limited As accumulation in rice grain and As detoxification, which is partially attributed to the indirectly altered expression of genes such as *OsPT1*, *OsPT4*, *OsNIP3;2*, *OsLsi6* and *OsOASTL-A1*. ROS homeostasis likely contributes to the regulation of As transport and tolerance mediated by OsAOS1 but not OsAOS2. Increased As sensitivity in the loss-of-function lines of *OsAOS2* is largely depended on the changed expression of *OsPT8*, *OsNIP3;2*, *OsLsi6* and *OsOASTL-A1*

In summary, our results indicate that OsAOS2 is important for As tolerance, while OsAOS1 confers both As allocation and detoxification, which could be partially attributed to the indirect effects of altered gene expression profiling and ROS homeostasis (Fig. 9).

## Materials and methods

### Plant materials and growth conditions

The mutant lines of *OsAOS1* and *OsAOS2* generated through CRISPR/Cas9 were obtained from the commercial rice genome-scale mutagenesis library, Biogle Genome Editing Center (Lu et al. 2017). The homozygous mutant lines, *osaos1–1* and *osaos2–1* were isolated through the sequencing results of the amplified DNA fragments containing the target sites of each candidate genes. Primers used in the study are listed in Table S1.

The two mutant lines together with the corresponding wild-type rice Zhonghua 11 (ZH11, *Oryza sativa ssp. Japonica*) were cultured in hydroponic condition or paddy soil for the phenotypic analyses. Briefly, rice seeds of each genotype were soaked in tap water at 37 °C for 2–3 d for germination, the seedlings were transferred to a net floating on a 0.5 mM CaCl_2_ solution for 3 days. After about 3–4 d, the plants were moved to the pots (13 L) containing one-half-strength Kimura B solution (Deng et al. 2013) for 6 d, and then the plants were transferred to smaller pots (4.2 L) for the further treatment. The nutrition solution was renewed every 2 d. The hydroponic solution was replaced by one-half-strength Kimura B solution without phosphorus (P) for 2 d and then followed with As-contained low-phosphorus (1 μM P) solution when the plants were treated with As(V) treatment. For treatment of plants with As(III), the required As concentrations were added directly to the one-half-strength Kimura B solution. The concentration of phosphorus in the normal one-half-strength Kimura B solution is 90 μM.

For hydroponic culture, the plants were grown in a growth chamber at 25–30 °C under long-day condition (16-h/8-h day/night photoperiod). The concentrations of As(III) and As(V) employed for the treatments were decided by the growth stage, the organs of the plants which we mainly focus on, as well as their varied toxicity. Three concentrations of phosphorus (P) were employed under different conditions in this study, the plants without As(V) treatments were cultured with half-strength Kimura B solution (90 μM P), before As(V) treatment, the plants were pre-cultured with half-strength Kimura B solution without P, and if the treatment with As(V) last for more than two days, the final P concentration in the solution was 1 μM.

For soil culture, the plants were grown in the paddy soil-contained pots (30 L) until fully mature under normal condition since May to August of 2022, which were conducted in the west campus of Yangtze University located in Jingzhou, Hubei Province, China. The hydro-soluble As concentration in the soil was about 2 mg/kg DW.

### Bioinformatics analysis of OsAOSs

The phylogenetic analysis of AOS members was performed according to our previous reports (Deng et al. 2022; Jiang et al. 2023). Briefly, the homologues of AOS from representative plant and algal species were identified through BLASTP searches from the OneKP database (https://db.cngb.org/blast/blast/blastp/) using OsAOS1 (OsAOS2, Supplementary Fig. 1) as the reference, the candidate sequences that satisfied the criteria, E value < 10^−10^ and query coverage > 50% were chosen for the further analysis. The phylogenetic tree was generated and displayed by CIPRES Science Gateway V 3.3 (https://www.phylo.org) and iTOL 6.0 visualization (https://itol.embl.de/), respectively. Furthermore, the multiple alignments of AOS1 homologues were conducted using MAFFT with the default setting (https://mafft.cbrc.jp/alignment/server/), and the 3D structure was predicted via SWISS-MODEL (https://swissmodel.expasy.org/interactive).

### Gene expression pattern analysis

To investigate the expression pattern of *OsAOS* genes in various organs, the rice plants (cv. Nipponbare) grown in paddy soil until grain filling stage, three independent samples of root, node II, Node I, internode II, internode I, leaf blade II, leaf blade I, rachis, and spikelet were sampled for RNA extraction and transcriptomic analysis (Personal Biotechnology Co., Shanghai, China). To examine the response of *OsAOS* genes to As(III) and 25 μM As(V), the datasheets derived from the previous studies were employed (Huang et al. 2012; Yu et al. 2012; Huang et al. 2019). To understand the expression pattern of *OsAOS* genes under As(V) conditions, the 0–2 cm length from the tip of seminal roots of WT (ZH11, 2 d old) seedlings subjected to 3 μM As(V) for 1, 6 and 24 hours were sliced for RNA-sequencing (Personal Biotechnology Co., Shanghai, China). The mean value of three FPKM (Fragments Per Kilobase of exon model per Million mapped fragments) of each gene (Fig. 1A), or the fold change compared to those under control conditions (Fig. 1B, C) were calculated for the generation of heat map, which were displayed by using TBtools (Chen et al. 2020).

### Phenotypic analysis at vegetative and reproductive stages

Two concentrations (2 and 5 μM) of As(III) [NaAsO_2_] and As(V) [Na_2_HAsO_4_] respectively were added to the hydroponic solutions for the determination of As accumulation 3 to 4-leaf stages in the tissues *osaos1–1*, *osaos2–1* and WT plants. The nutrient solution was renewed every 2 d. The growth parameters of each plant including plant height were recorded every 2 d. After 6 d of As treatments, the leaves and the roots were harvested after being washed with pre-chilled 0.5 mM CaCl_2_ solution for 3 times. The fresh weight (FW, mg) of each sample was measured and then dried at 65 °C for 3 d.

For As tolerance investigation, 10-d-old seedlings of the three genotypes were treated with 2, 5, and 10 μM As(V)-contained one-half-strength Kimura B solution with 1 μM P for 10 d, and then the samples were harvested and their dry weight were measured. Four and eight biological replicates were used for dry weight and plant height, respectively. The experiments were repeated at least twice.

Five plants of each genotype (*osaos1–1*, *osaos2–1*, and WT) were grown in soil in a 30-L pot with 5 replicates for each genotype. At harvested, the seed setting ratio were calculated according to our previous study by using 8.5% NaCl solution for selection (Deng et al. 2013). The grain yield per plant and the weight per 1000 seeds were recorded. Brown rice sets from each plant were randomly selected for further digestion and metal measurement.

### Determination of metals in plant tissues and xylem sap

For xylem sap collection, 30-day-old seedlings of *osaos1–1*, *osaos2–1*, and WT were employed. The hydroponic solution was based on one-half-strength Kimura B solution without P for 2 d, and then 2 μM As(V) was added into the pots and mixed well with glass rod, xylem sap was collected after the plants were treated for 30 mins with a pipette for 0.5 h (Deng et al. 2013; Tang et al. 2019). Five independent samples from each genotype and treatment were collected and then diluted with 2% HNO_3_ for metal concentration determination by inductively coupled plasma-mass spectrometry (ICP-MS, NexION 1000; PerkinElmer). The dried shoot, root, basal region, and brown rice without husk was digested with concentrated HNO_3_ at a temperature up to 140 °C. The metal concentrations in the digested solution were determined by Inductively Coupled Plasma-Mass Spectrometry (ICP-MS) after dilution with MQ water (Cheng et al. 2023; Ning et al. 2023).

### Measurement of As efflux activity

For As efflux activity test, the germinated seeds of each genotype (*osaos1–1*, *osaos2–1*, and WT) were grown in one-half-strength Kimura B solution until 12-d-old, and then the plants were pre-treated one-half-strength Kimura B without P for 2 days. The plants were moved in to the 5 μM As(V)-contained one-half-strength Kimura B solution (−P) for 4 hours, the roots were washed with 0.5 mM CaCl_2_ and the plants were transferred into 110 mL black bottles with one-half-strength Kimura B solution (−P) for efflux. After 6 h, 1 mL solution was collected for As concentration measurement, the roots and shoots were harvested after being washed with pre-cold 0.5 mM CaCl_2_ solution for 3 times. One plant for one black bottle, and 5 replicates were set for each genotype in this experiment. As efflux ratio was calculated as the following formula: As in solution/[As in solution + As in root + As in shoot] × 100%.

### Relative root elongation under As

To compare the As tolerance of *osaos1–1*, *osaos2–1*, and WT, 2-day-old seedlings were exposed to a 0.5 mM CaCl_2_ solution (pH 5.6) containing 15 μM As(III) or 2 μM As(V) for 2 d. The root length of each seedling was measured with a ruler before and after the treatments for 24 and 48 h, and the relative root elongation (root elongation with As/root elongation without As × 100%) was calculated. Fifteen replicates were conducted for each treatment, and the experiments were repeated at least twice.

### RNA extraction and quantitative real-time PCR

The roots and shoots of *osaos1–1*, *osaos2–1*, and WT subjected to 2 or 5 μM As(V) for 6 d were harvested for RNA extraction, which is following the user manual of TRIpure Reagent (Aidlab). The first strand of cDNA synthesized using the TRUEScript RT Kit (+ gDNA Eraser) reverse transcription kit (Aidlab) was employed as the template. Quantitative real-time PCR (qPCR) with 3 biological replicates was performed according to our previous report (Jiang et al. 2023). Ct values were normalized to the corresponding endogenous control gene (*OsActin1,* LOC_Os03g50885), the relative expression levels of the genes involved in As uptake, translocation, and detoxification and JA-responsive marker genes were calculated using the ΔΔ Ct method (Huang et al. 2016). The primers used in this study are shown in Supplemental Table S1.

### Reactive oxygen species detection

After germination for 3 d on the floating net with the supplement of 0.5 mM CaCl_2_, the seedlings of *osaos1–1*, *osaos2–1*, and WT were treated with 2 μM As(V). To detect the accumulation of ROS, the roots of the plants subjected to As(V) for 1 and 6 h were stained with 10 μM ROS indicator CM-H_2_DCFDA (C6827, Thermo Fisher Scientific) in PBS solution for 1 h under dark condition. The green fluorescent signal in the root region was observed after being washed with PBS for 3 times and the photos were taken with a fluorescent microscope. The plants without As(V) treatment were employed as controls. The intensity of the signal was captured and calculated with the software ImageJ (version 1.8.0, National Institutes of Health, USA) (Cheng et al. 2023). Five seedlings were used for each treatment.

### Data analysis

Data analysis was conducted by using SPSS software, with the statistical significance analysis performed using one-way analysis of variance (ANOVA) or the least significant difference (LSD) test for mean comparison (at the 5% level).

### Supplementary Information

**Additional file 1: Supplemental Table S1.** Primers used in this study.

**Additional file 2: Supplementary Fig. 1.** Evolutionary analysis of AOS2 homologues in land plants and algal species. **Supplementary Fig. 2.** CRISPR/Cas9-induced mutations of *OsAOS1*. **Supplementary Fig. 3.** CRISPR/Cas9-induced mutations of *OsAOS2*. **Supplementary Fig. 4.** As accumulation and distribution in the seedlings of *osaos1–1*, *osaos2–1*, and WT subjected to As(III) for 6 days. **Supplementary Fig. 5.** As efflux activity of *osaos1–1*, *osaos2–1* and WT. **Supplementary Fig. 6.** Biomass of *osaos1–1*, *osaos2–1* and WT subjected to 2 or 5 μM As(V) for 6 days. **Supplementary Fig. 7.** Plant growth and biomass of *osaos1–1*, *osaos2–1* and WT subjected to 2 or 5 μM As(III) for 6 days.

## Data Availability

The author responsible for distribution of materials integral to the findings presented in this article is Fenglin Deng (dfl@yangtzeu.edu.cn).

## Abbreviations

AOS: Allene oxide synthase
As(III): Arsenite
As(V): Arsenate
As: Arsenic
CM-H_2_DCFDA: 5-(and-6)-chloromethyl-2′, 7′-dichlorodihydrofluorescein diacetate, acetyl ester
CRISPR/Cas9: Clustered regularly interspaced short palindromic repeats–associated nuclease 9
ICP-MS: Inductively coupled plasma-mass spectrometry
JA: Jasmonic acid
JA-Ile: Jasmonoyl-l-isoleucine
MeJA: Methyl jasmonate
