# Supplementary material for: Allene oxide synthase 1 contributes to limiting grain arsenic accumulation and seedling detoxification in rice
Source: Stress Biol. 2023 Nov 30;3(1):52. doi: 10.1007/s44154-023-00136-8 (PMC10689621; doi:10.1007/s44154-023-00136-8)
Supplement: Supplementary file 1 — Additional file 1: Supplemental Table S1. Primers used in this study. [file 44154_2023_136_MOESM1_ESM.doc]

Supplemental Table S1. Primers used in this study.

| **Primer ID** | **Primer sequence**(5’-3’) | **Objective** |
| --- | --- | --- |
| OsActin-F | GACTCTGGTGATGGTGTCAGC | qRT-PCR |
| OsActin-R | GGCTGGAAGAGGACCTCAGG | qRT-PCR |
| OsLsi1-F | cggtggatgtgatcggaacca | qRT-PCR |
| OsLsi1-R | cgtcgaacttgttgctcgcca | qRT-PCR |
| OsLsi2-F | ATCTGGGACTTCATGGCCC | qRT-PCR |
| OsLsi2-R | ACGTTTGATGCGAGGTTGG | qRT-PCR |
| OsLsi3-F | CTGTATCCCTGTTGCCAGCTG | qRT-PCR |
| OsLsi3-R | TAATCCGGCATGCGTACTTG | qRT-PCR |
| OsLsi6-F | GAGTTCGACAACGTCTAATCGC | qRT-PCR |
| OsLsi6-R | AGTACACGGTACATGTATACACG | qRT-PCR |
| OsABCC1-F | AACAGTGGCTTATGTTCCTCAAG | qRT-PCR |
| OsABCC1-R | AACTCCTCTTTCTCCAATCTCTG | qRT-PCR |
| OsABCC7-F | TAAAGCAGCCTTCCTTCTCA | qRT-PCR |
| OsABCC7-R | GCCTTCGCACCTACAAATAC | qRT-PCR |
| OsPT1-F | GGAGCGTTCGGGTTCCTGT | qRT-PCR |
| OsPT1-R | GGCGACCTCCTGCGAGATAA | qRT-PCR |
| OsPT4-F | TTCTGCTAGTGTACCAAACAAAATTACA | qRT-PCR |
| OsPT4-R | GTAAGTGGCATTTATAATATCAACAGTAACC | qRT-PCR |
| OsPT8-F | GCCAAGAACGCCAAGCAGGC | qRT-PCR |
| OsPT8-R | GGAGAAGAGGCCGAAGCTGC | qRT-PCR |
| OsHAC1;1-F | GCAGCACCGATGAGATGAGC | qRT-PCR |
| OsHAC1;1-R | TGCCTCCATAATACCAGTAGTAACAAAC | qRT-PCR |
| OsHAC1;2-F | TAGCATCTGCCGATCTCATA | qRT-PCR |
| OsHAC1;2-R | GAGGTTTATTCACCGCAAGG | qRT-PCR |
| OsHAC4-F | TGCTCGCTATCCGGGAGATG | qRT-PCR |
| OsHAC4-R | GCGGCAGATGAGTACAACAGAA | qRT-PCR |
| OsNIP3;2-F | CCAGTAGACCTCCCAAAGATAA | qRT-PCR |
| OsNIP3;2-R | GTGCAGCAATGTAAGGAAGAAG | qRT-PCR |
| OsOASTL-A1-F | GATGCCTAACTCTTACATTCTCC | qRT-PCR |
| OsOASTL-A1-R | CTTTCTGTCGGTTCAACACC | qRT-PCR |
| OsAOS1-F | TGTGGACTTGCAGTTGGAGC | mutant identification |
| OsAOS1-R | CATGAAGGTGCCGGTGAAGA | mutant identification |
| OsAOS2-F | CACGGGCTATTTCTACCCCC | mutant identification |
| OsAOS2-R | CATGAACGTGCCGGTGAAC | mutant identification |
