# Supplementary material for: Allene oxide synthase 1 contributes to limiting grain arsenic accumulation and seedling detoxification in rice
Source: Stress Biol. 2023 Nov 30;3(1):52. doi: 10.1007/s44154-023-00136-8 (PMC10689621; doi:10.1007/s44154-023-00136-8)
Supplement: Supplementary file 2 — Additional file 2: Supplementary Fig. 1. Evolutionary analysis of AOS2 homologues in land plants and algal species. Supplementary Fig. 2. CRISPR/Cas9-induced mutations of OsAOS1. Supplementary Fig. 3. CRISPR/Cas9-induced mutations of OsAOS2. Supplementary Fig. 4. As accumulation and distribution in the seedlings of osaos1–1, osaos2–1, and WT subjected to As(III) for 6 days. Supplementary Fig. 5. As efflux activity of osaos1–1, osaos2–1 and WT. Supplementary Fig. 6. Biomass of osaos1–1, osaos2–1 and WT subjected to 2 or 5 μM As(V) for 6 days. Supplementary Fig. 7. Plant growth and biomass of osaos1–1, osaos2–1 and WT subjected to 2 or 5 μM As(III) for 6 days. [file 44154_2023_136_MOESM2_ESM.pptx]

## Slide 1
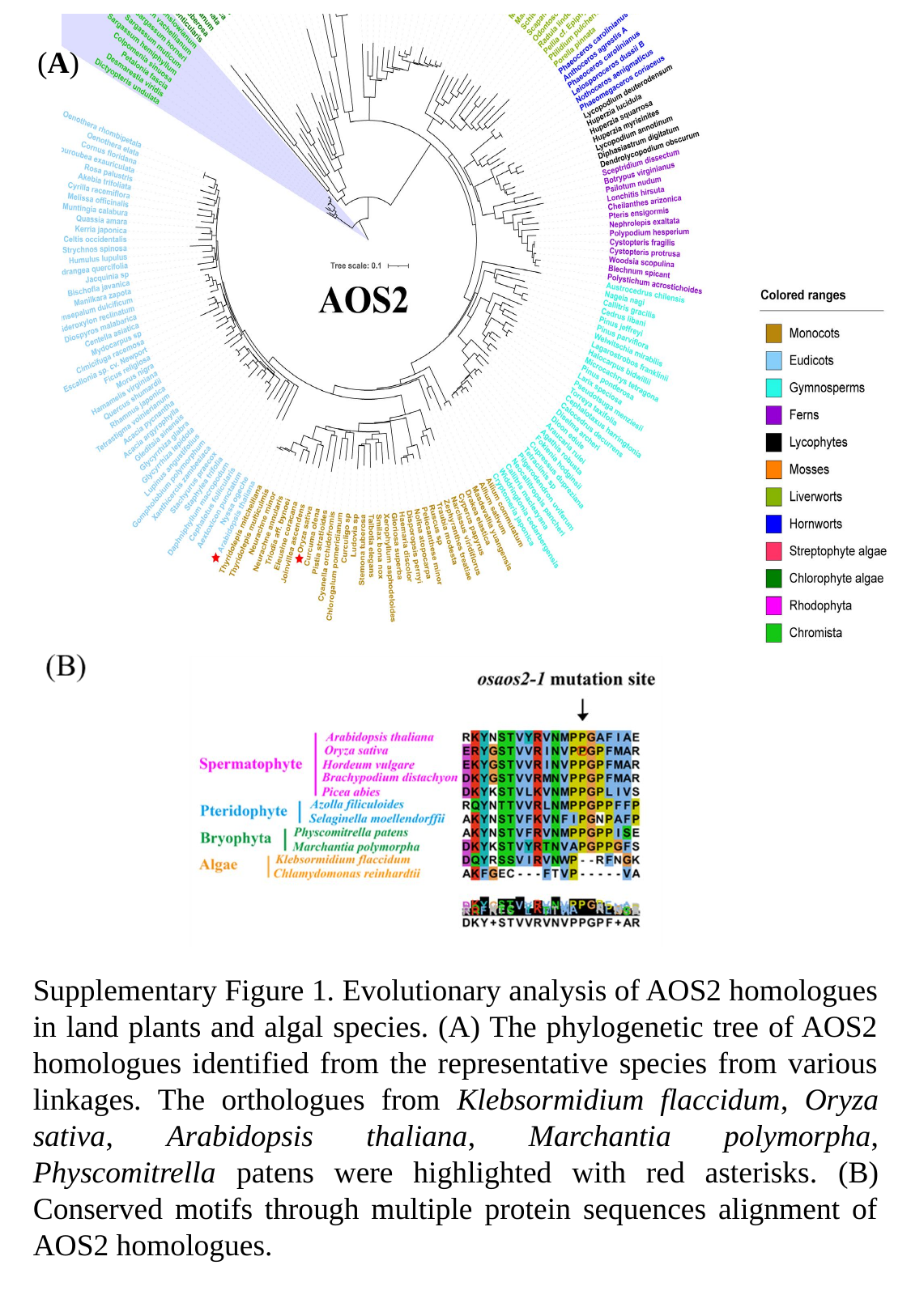

(A)
Supplementary Figure 1. Evolutionary analysis of AOS2 homologues in land plants and algal species. (A) The phylogenetic tree of AOS2 homologues identified from the representative species from various linkages. The orthologues from Klebsormidium flaccidum, Oryza sativa, Arabidopsis thaliana, Marchantia polymorpha, Physcomitrella patens were highlighted with red asterisks. (B) Conserved motifs through multiple protein sequences alignment of AOS2 homologues.

## Slide 2
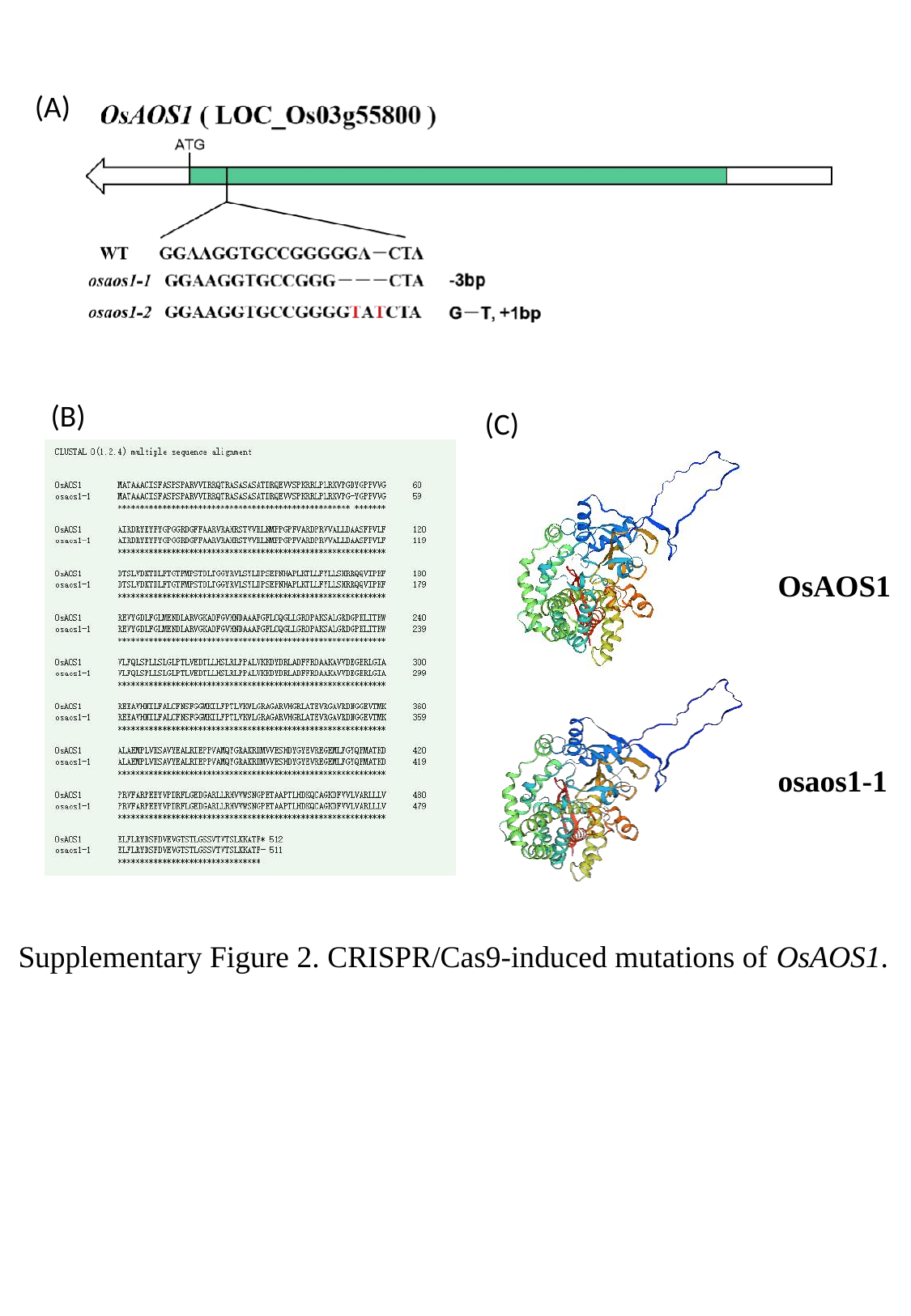

(A)
(B)
(C)
OsAOS1
osaos1-1
Supplementary Figure 2. CRISPR/Cas9-induced mutations of OsAOS1.

## Slide 3
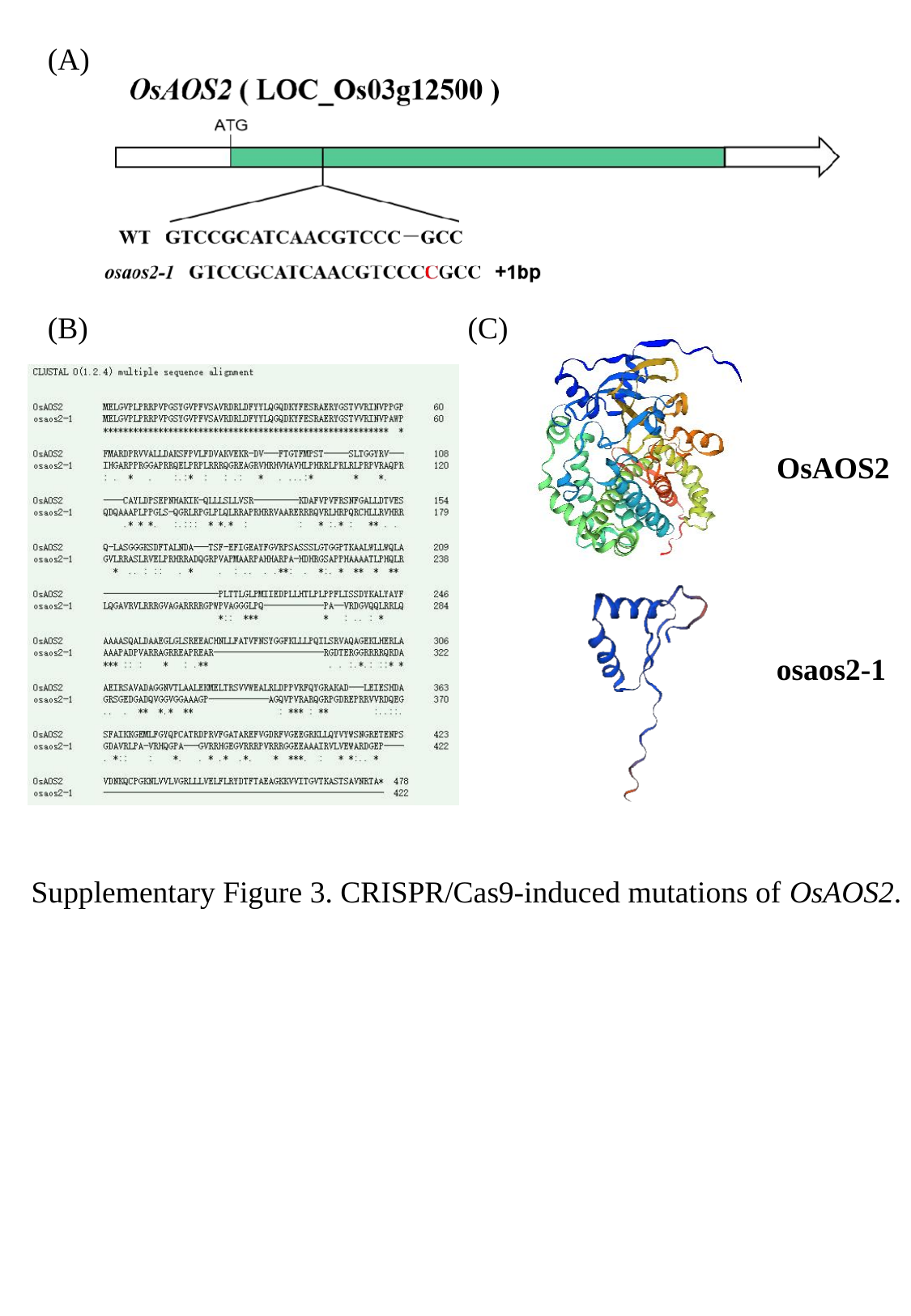

(A)
(B)
(C)
OsAOS2
osaos2-1
Supplementary Figure 3. CRISPR/Cas9-induced mutations of OsAOS2.

## Slide 4
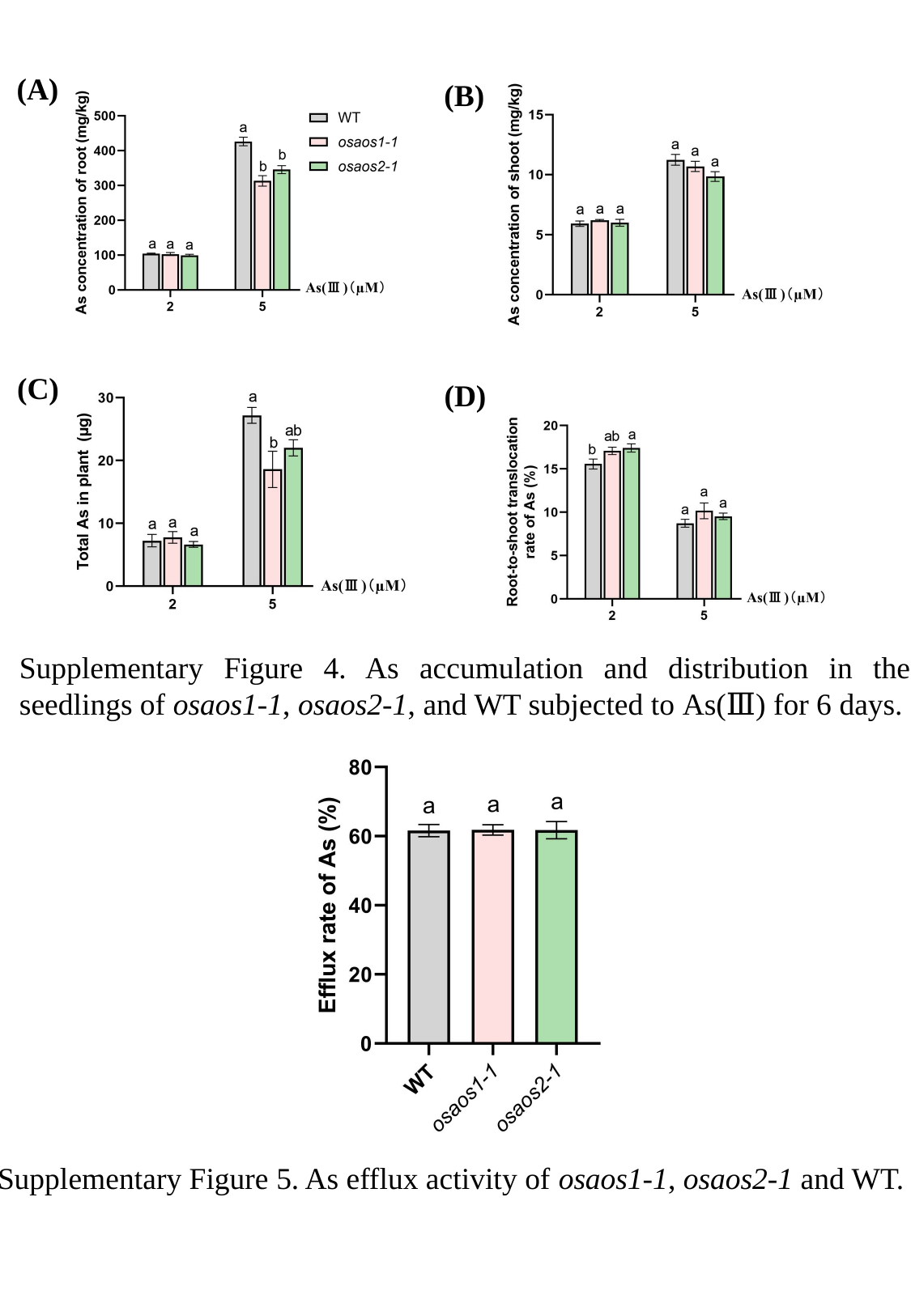

(A)
(B)
(C)
(D)
Supplementary Figure 4. As accumulation and distribution in the seedlings of osaos1-1, osaos2-1, and WT subjected to As(Ⅲ) for 6 days.
Supplementary Figure 5. As efflux activity of osaos1-1, osaos2-1 and WT.

## Slide 5
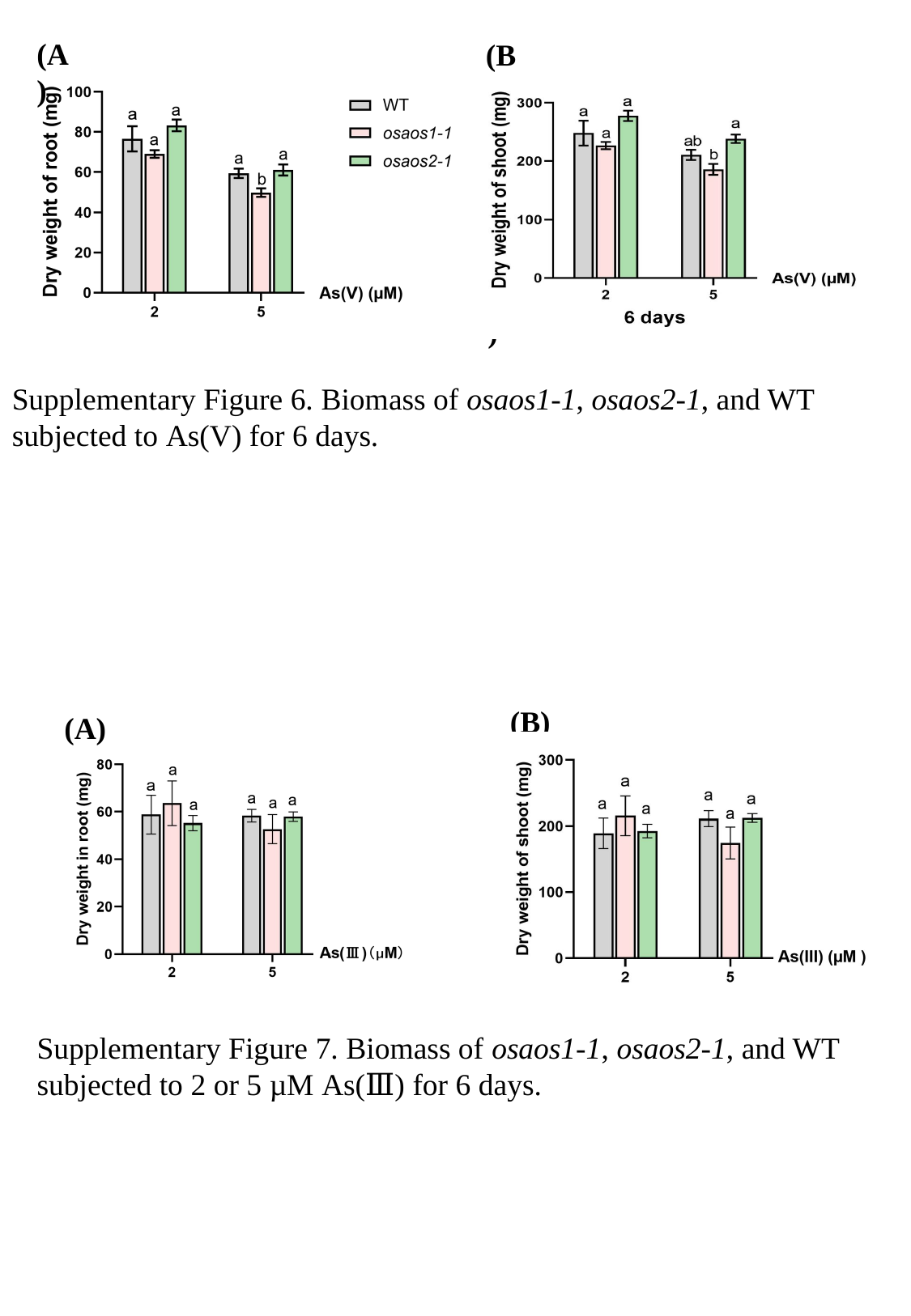

(A)
(B)
(C)
Supplementary Figure 6. Biomass of osaos1-1, osaos2-1, and WT subjected to As(V) for 6 days.
(B)
(A)
Supplementary Figure 7. Biomass of osaos1-1, osaos2-1, and WT subjected to 2 or 5 µM As(Ⅲ) for 6 days.

## Slide 6
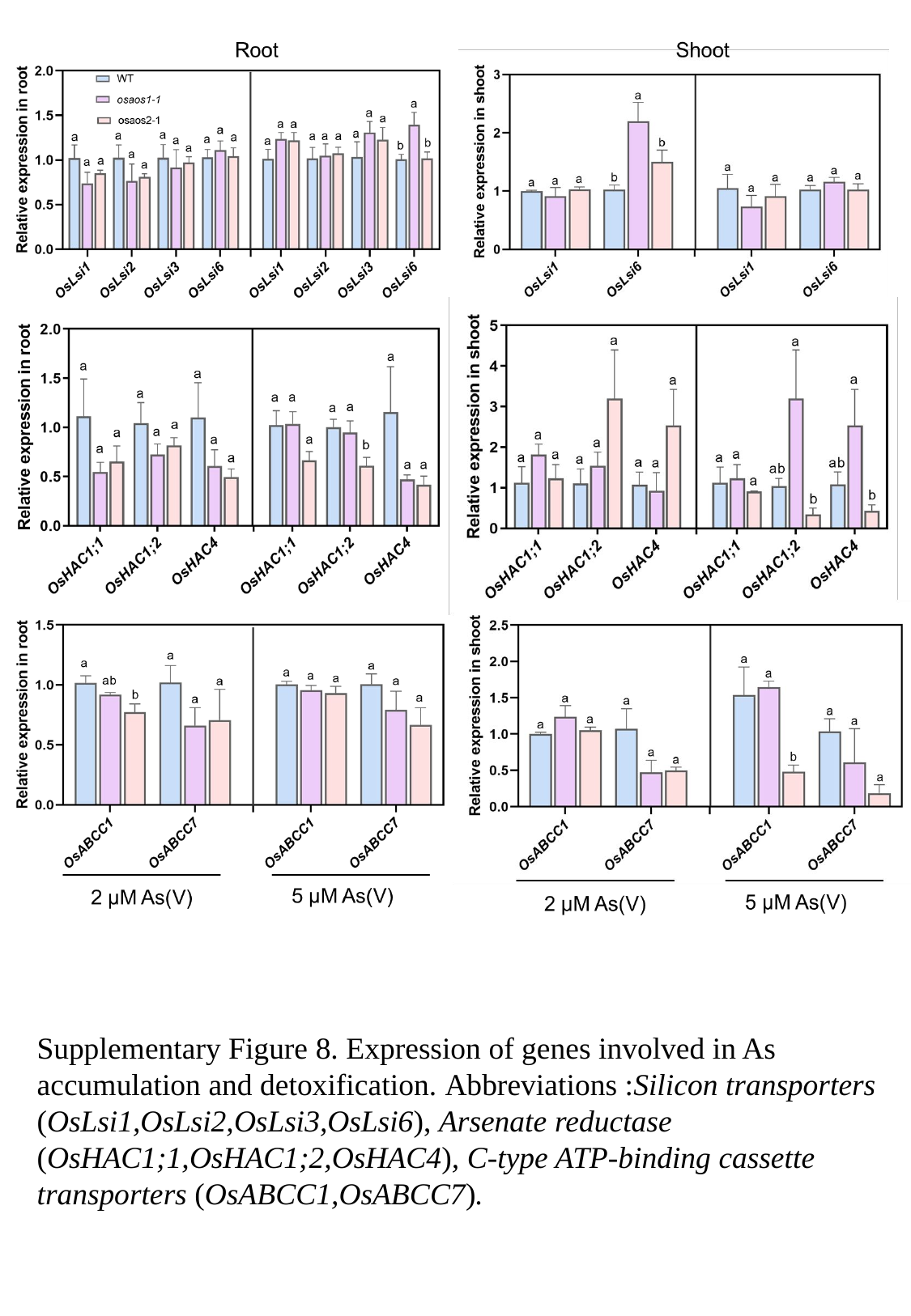

Supplementary Figure 8. Expression of genes involved in As accumulation and detoxification. Abbreviations :Silicon transporters (OsLsi1,OsLsi2,OsLsi3,OsLsi6), Arsenate reductase (OsHAC1;1,OsHAC1;2,OsHAC4), C-type ATP-binding cassette transporters (OsABCC1,OsABCC7).
